# Quantifying coupling and causality in dynamic bivariate systems: a unified framework for time-domain, spectral, and information-theoretic analysis

**DOI:** 10.3389/fnetp.2025.1687132

**Published:** 2026-01-06

**Authors:** Laura Sparacino, Helder Pinto, Chiara Barà, Yuri Antonacci, Riccardo Pernice, Ana Paula Rocha, Luca Faes

**Affiliations:** 1 Biosignals and Information Theory Laboratory, Department of Engineering, University of Palermo, Palermo, Italy; 2 CMUP LASI, Departamento de Matemática, Faculdade de Ciências, Universidade do Porto, Porto, Portugal; 3 Faculty of Technical Sciences, University of Novi Sad, Novi Sad, Serbia

**Keywords:** correlation, causality, information dynamics, spectral integration property, parametric autoregressive models, network physiology

## Abstract

Understanding the underlying dynamics of complex real-world systems, such as neurophysiological and climate systems, requires quantifying the functional interactions between the system units under different scenarios. This tutorial paper offers a comprehensive description to time, frequency and information-theoretic domain measures for assessing the interdependence between pairs of time series describing the dynamical activities of physical systems, supporting flexible and robust analyses of statistical dependencies and directional relationships. Classical time and frequency domain correlation-based measures, as well as directional approaches derived from the notion of Granger causality, are introduced and discussed, along with information-theoretic measures of symmetrical and directional coupling. Both linear model-based and non-linear model-free estimation approaches are thoroughly described, the latter including binning, permutation, and nearest-neighbour estimators. Special emphasis is placed on the description of a unified framework that establishes a connection between causal and symmetric, as well as spectral and information-theoretic measures. This framework enables the frequency-specific representation of information-theoretic metrics, allowing for a detailed investigation of oscillatory components in bivariate systems. The practical computation of the interaction measures is favoured by presenting a software toolbox and two exemplary applications to cardiovascular and climate data. By bridging theoretical concepts with practical tools, this work enables researchers to effectively investigate a wide range of dynamical behaviours in various real-world scenarios in Network Physiology and beyond.

## 1 Introduction

A central challenge in the broad field of Network Science consists in deciphering how complex, system-wide dynamics emerge from the interactions among the units of a distributed network (Bashan et al., 2012; Barabási, 2013; Bassett and Sporns, 2017). A key strategy to tackle this problem involves quantifying bivariate interactions, that is, measuring how pairs of individual components influence each other over time. Numerous data-driven methodologies for network inference have been developed to investigate interactions emerging from time-resolved data, enabling researchers to map the functional interdependencies that drive the dynamic behaviours of the investigated systems. For instance, in neuroscience, functional connectivity between pairs of brain regions has often been assessed via correlation-based measures, capturing co-activation patterns linked to cognition and altered in pathological conditions such as Alzheimer and schizophrenia (Buckner et al., 2005; Bassett and Sporns, 2017). In regulatory physiology, the dynamic activity of cardiovascular, cardiorespiratory and cerebrovascular systems has been widely investigated using dynamic measures of complexity and causality in different experimental conditions and patho-physiological states, evidencing well-known behaviours including frequency-specific responses along the baroreflex and the pressure-to-flow link [see, e.g., Schulz et al. (2013); Bari et al. (2016); Porta and Faes (2015); Pernice et al. (2022b); Sparacino et al. (2024a); Cairo et al. (2025)]. Also in very different fields such as earth system science, causal approaches have revealed the presence and importance of directional links, e.g., between sea-surface temperature and fish populations (Sugihara et al., 2012), as well as between atmospheric patterns and air circulation (Runge et al., 2019).

In the context of data-driven network modelling, bivariate interactions have been inferred from pairs of time series describing the dynamic activities of the two investigated systems, and generally assessed by measures of coupling and causality in the time, frequency and/or information-theoretic domains (Pereda et al., 2005; Porta and Faes, 2015; Schulz et al., 2013; Edinburgh et al., 2021; Cliff et al., 2021). Specifically, *non-directional coupling* relations between time series refer to associations which do not specify the direction of influence, rather look for symmetrical statistical dependencies between them (Faes et al., 2012a; Faes and Nollo, 2013). A common and simple approach to compute non-directional coupling in the time domain is cross-correlation, which measures the similarity between time series as a function of the time lag, capturing synchronous or time-shifted dependencies (Reinsel, 2003; Pereda et al., 2005). While straightforward and computationally efficient, cross-correlation assumes linearity, can be sensitive to noise or temporal misalignment (Cliff et al., 2021) and does not assume causality between the time series. Nevertheless, the principle of *causality* is fundamental to identify driver-response (i.e., time-lagged) relations between the series. In the linear signal processing framework, this principle has been explored with reference to the concept of Granger causality (GC), originally developed by Wiener (Wiener, 1956) and then made operative by Granger in the context of linear regression models (Granger, 1969). In particular, GC relates the presence of a cause-effect relation to two aspects: the cause must precede the effect in time and must carry unique information about the present value of the effect. This relationship is not symmetrical and can be bidirectional, thus enabling the detection of both directional and reciprocal influences (Bressler and Seth, 2011; Porta and Faes, 2015). Differently from non-directional measures, causality approaches exploiting this concept have allowed focusing on specific directional pathways of interaction within the investigated network, as widely done, e.g., in neurophysiology (Ding et al., 2006; Porta and Faes, 2015; Seth et al., 2015; Koutlis et al., 2021; Charleston-Villalobos et al., 2023; Pernice et al., 2022a; Difrancesco et al., 2023; Pichot et al., 2024), or in climate (Smirnov and Mokhov, 2009; McGraw and Barnes, 2018; Runge et al., 2019) and ecological (Freeman, 1983; Sugihara et al., 2012) sciences.

A limitation of traditional time domain measures of coupling and causality is their lack of frequency resolution, as they capture overall dependencies between two time series without isolating contributions from specific oscillatory components. This represents an issue in fields such as cardiovascular analysis, where physiological signals including heart rate and blood pressure exhibit distinct rhythmic patterns, especially within the low-frequency (LF, 0.04−0.15 Hz) and high-frequency (HF, 0.15−0.4 Hz) bands (Cohen and Taylor, 2002). To overcome this issue, representations of coupling and causality in the frequency domain are often desirable to examine oscillatory interactions and identify the individual rhythmic components in the measured data. For this purpose, linear parametric model-based and non-parametric approaches, the latter based on the definition of the cross power spectral density as the Fourier transform of the cross-correlation function (Priestley, 1981), have been widely used to represent signals in the frequency domain. For instance, power spectral density estimates and spectral coherence (Kay, 1988), which is the frequency domain counterpart of the time domain cross-correlation, have been adopted to study a variety of patho-physiological conditions in brain (Möller et al., 2001; Coben et al., 2008; Milde et al., 2010; Wang et al., 2015), cardiovascular (Orini et al., 2011; Daoud et al., 2018) and climate (Antonacci et al., 2021b) networks. Additionally, spectral causality measures including directed coherence (Saito and Harashima, 1981; Baccalá et al., 1998) and linear feedback (Geweke, 1982) have been defined to explore frequency-specific directional patterns, and applied to a variety of neurophysiological data (Brovelli et al., 2004; Faes et al., 2005; 2012a; Barrett et al., 2012; Porta and Faes, 2013; Pernice et al., 2022b; Clemson et al., 2022).

Statistical dependencies among real-world time series can be evaluated using information-theoretic tools. Entropy-based measures of mutual information, mutual information rate and transfer entropy have been largely exploited to assess the overall information shared between two interdependent systems (Shannon, 1948; Cover, 1999), the dynamic interdependence between two systems per unit of time (Gelfand and Yaglom, 1959; Duncan, 1970; Faes et al., 2022; Barà et al., 2023; Antonacci et al., 2025; Pinto et al., 2025a), and the dynamic information transferred to a selected target system from the other connected system (Schreiber, 2000; Wibral et al., 2014; Faes et al., 2015b; 2016a; Shao et al., 2023), respectively. Remarkably, the mutual information rate represents a dynamic version of the mutual information and, as such, reflects the strength of the symmetrical statistical association between two dynamic systems. It has been decomposed into terms with meaningful physical interpretations corresponding to the well-known conditional entropy and transfer entropy measures (Barà et al., 2023; Pinto et al., 2025a). These metrics are closely related to the notions of complexity of individual systems and causality between pairs of systems, and widely exploited in various applications to real data (Faes et al., 2013c; Runge et al., 2019; Martins et al., 2020; Silini et al., 2023; Barà et al., 2023).

Information-theoretic measures have the main advantage of generality, being defined in terms of probability distributions, and can thus be stated in a fully model-free formulation (Ash, 2012). Examples of model-free estimators include the *k*-nearest neighbour (Kozachenko and Leonenko, 1987; Kraskov et al., 2004), permutation-based (Bandt and Pompe, 2002; Barà et al., 2023) and binning (Darbellay and Vajda, 1999; Cellucci et al., 2005; Azami et al., 2023) approaches. These methods enable the detection of non-linear and consequently more complex relationships, although they require trade-offs in terms of dimensionality, estimation accuracy, computational complexity and sensitivity to parameter choices. Nevertheless, information measures can also be expressed in terms of predictability improvement under the two key assumptions of linearity and joint Gaussianity; if this is the case, their computation relies on parametric autoregressive models (Lütkepohl, 2005; Barnett et al., 2009), whereby concepts of prediction error and conditional entropy, GC and transfer entropy, or spectral coherence and mutual information rate, have been linked to each other in the time and frequency domains (Geweke, 1982; Barnett et al., 2009; Barrett et al., 2010; Chicharro, 2011; Faes et al., 2015b; Porta and Faes, 2015; Faes et al., 2016b). Remarkably, the information-theoretic and spectral formulations are tightly connected thanks to the fulfillment of the spectral integration property, which is essential to allow quantification of these measures with reference to specific oscillatory components contained within spectral bands of interest.

In the wide context of bivariate time series analysis, this work presents a coherent theoretical framework in which measures of coupling and causality in the time, frequency, and information-theoretic domains are thoroughly reviewed and described, emphasizing properties and relations across domains (Section 1). The practical implementation of the measures is favoured by the exploitation of parametric autoregressive models (Section 2), which establish a connection between information-theoretic and spectral formulations under the assumptions of linearity and joint Gaussianity, and model-free approaches (Section 3), including techniques such as coarse-graining of embedding spaces (*k*-nearest neighbours estimator) or symbolic representations of the observed dynamics (binning and permutation estimators). The software and the codes relevant to the practical computation and statistical validation of the bivariate interaction measures are presented in Section 4, and collected in the BIM (Bivariate information measures) Matlab toolbox available for free download from https://github.com/laurasparacino. To illustrate the behaviour of the discussed measures and to showcase their implementation allowed by the BIM toolbox, illustrative examples are finally reported regarding benchmark applications to cardiovascular and climate time series (Section 5).

## 2 Framework for the analysis of interactions in bivariate systems

**FIGURE 1:**
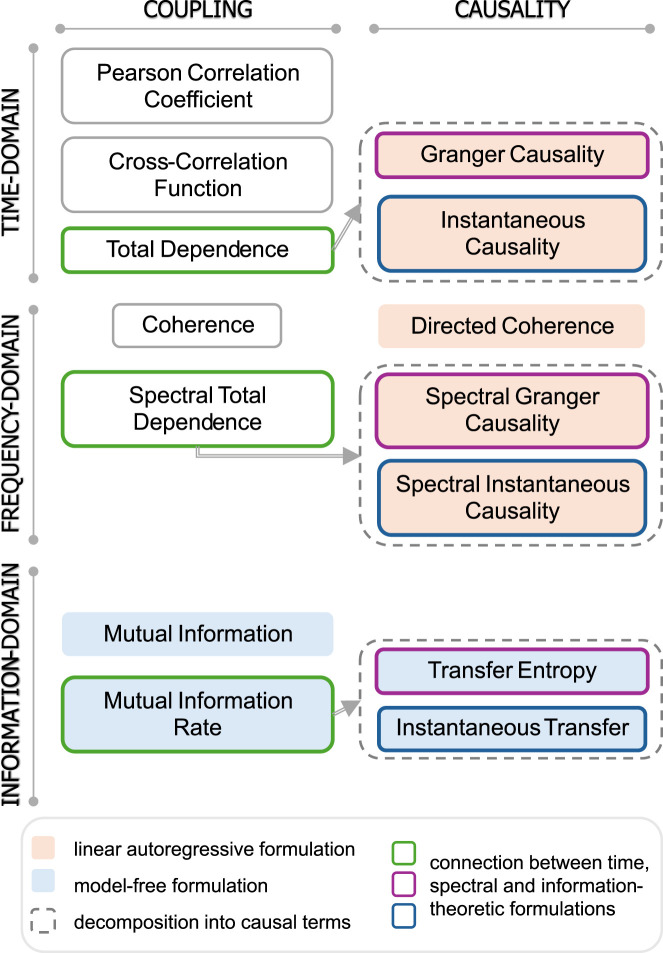
Overview of coupling and causality measures across time, frequency, and information-theoretic domains, as reviewed and discussed in this work. The implementation through linear autoregressive models and model-free approaches is discussed in Section 2 and Section 3, respectively, while the mathematical connection between formulations in the three domains is presented in Section 2.5.

This section first introduces basic concepts of probability related to static and dynamic systems (Section 1.1) (Papoulis and Pillai, 2002). Then, it illustrates well-known correlation-related measures in the time and frequency domains (Section 1.2), as well as measures that implement the concepts of coupling and causality applied to random variables and processes in the field of information-theory (Section 1.3). A schematic overview of these measures, emphasizing the nature (coupling vs. causality) and computation domain (time, frequency, information-theoretic) of the measures as well as their conceptual and mathematical links, is given in Figure 1.

### 2.1 Basic notions of probability

#### 2.1.1 Static analysis of random variables

A *random variable* is a mathematical variable whose value is subject to variations due to chance. Specifically, continuous random variables can take values inside an infinite-dimensional set usually denoted as the domain. The generic scalar random variable V with domain $D_{V}$ is characterized by its distribution function, which assigns a probability to each measurable subset of $D_{V}$ . Formally, the probability for the variable V of taking values within the interval $\left[a,b\right]\subseteq D_{V}$ is determined by the integral $Pr\left\{a\leq V\leq b\right\}=\int_{a}^{b}p_{V}\left(v\right)\text{d}v=F_{V}\left(b\right)-F_{V}\left(a\right)$ , where $p_{V}$ is the *probability density function* of the variable and $F_{V}$ is its cumulative distribution function. The cumulative distribution quantifies the probability that the variable V has v as its upper bound, $F_{V}\left(v\right)=Pr\left\{V\leq v\right\}$ , while the probability density is mathematically defined as the derivative of the cumulative distribution, in a way such that $F_{V}\left(v\right)=\int_{-\infty }^{v}p_{V}\left(u\right)\text{d}u$ . These definitions extend in a straightforward way to the generic 2-dimensional variable $\text{V}=\left\{V_{1},V_{2}\right\}$ by defining the *joint probability density function* $p_{V_{1},V_{2}}\left(v_{1},v_{2}\right)$ and performing multiple integration over the domain of each scalar component to get the cumulative distribution. Moreover, the *conditional probability density function* of, e.g., $V_{1}$ given $V_{2}$ expresses the probability of observing the value $v_{1}$ for $V_{1}$ given that the value $v_{2}$ has been observed for $V_{2}$ : $p_{V_{1}|V_{2}}\left(v_{1}|v_{2}\right)=\frac{p_{V_{1},V_{2}}\left(v_{1},v_{2}\right)}{p_{V_{2}}\left(v_{2}\right)}$ .

The bivariate interactions between the two variables $V_{1},V_{2}$ can be investigated by means of a *static analysis* of pairs of realizations of these variables available in the form of two sequences of data. Static analysis implicitly disregards temporal correlations, assuming that all samples of a data sequence are observation of the same single random variable and thus taking into account only zero-lag effects between the two data sequences analysed.

#### 2.1.2 Dynamic analysis of random processes

Contrarily to static systems, dynamic systems take values over diverse states at different instants of time, thus being explicitly dependent on the flow of time. The evolution over time of these systems can be only described in probabilistic terms using *random processes*, which can be thought of as sequences of random variables ordered according to time. Formally, the states visited by a generic dynamic system over time are described as a stochastic process $Y=\left\{Y_{n}\right\}$ , n=1,2,… , where the random variable $Y_{n}$ describes the $n^{\text{th}}$ state assumed by the system at the $n^{\text{th}}$ time step. Then, a realization of the stochastic process Y is the time series y={y(1),…,y(L)} , containing the values of Y collected over L time points. Setting a temporal reference frame in which n represents the present time, we denote as $Y_{n}$ the random variable describing the present state of Y , and as $Y_{n}^{-}=\left[Y_{n-1},Y_{n-2},\ldots \right]$ the random variable that samples the process over the whole past history. In general, the operation of separating the present from the past allows to consider the flow of time and to study the causal interactions within and between processes by looking at the statistical dependencies among these variables. In fact, the dynamic properties of a system are studied in statistics introducing the concept of *transition probability*, which is the probability associated with the transition of the system from its past states to its present state. Thus, the state transition of the history of Y relevant to the present state $Y_{n}$ is described by the conditional probability density $p_{Y_{n}|Y_{n}^{-}}\left(y_{n}|y_{n}^{-}\right)$ .

A useful property of stochastic processes is *wide-sense stationarity* (WSS), which defines the time-invariance of any joint probability density taken from the process. When the process is stationary, its composing variables are identically distributed, meaning that that the probability density is the same at all times; in practice, this allows to pool together the observations measured across time order to estimate the densities, thus enabling the estimation of probabilities from an individual realization of the process, i.e., a single time series (Faes et al., 2016b). For a stationary stochastic process, also the transition probabilities are time-independent, i.e., $p_{Y_{n}|Y_{n}^{-}}\left(y_{n}|y_{n}^{-}\right)=p_{Y}\left(y_{n}|y_{n}^{-}\right)$ . An important class of dynamic processes is represented by *Markov processes*, for which the present depends on the past only through a finite number of time steps. Specifically, the process Y is a Markov process of order q if its transition probability function satisfies the condition $p_{Y}\left(y_{n}|y_{n}^{-}\right)=p_{Y}\left(y_{n}|y_{n-1},y_{n-2},\ldots ,y_{n-q}\right)$ . With this notation, we define as $Y_{n}^{q}=\left[Y_{n-1},\ldots ,Y_{n-q}\right]$ the random variable that samples the process over the past q lags, with $Y_{n}^{-}=\lim_{q\to \infty }Y_{n}^{q}$ .

The bivariate interactions between two generic processes $\text{Y}=\left\{Y_{1},Y_{2}\right\}$ can be investigated by means of a *dynamic analysis*, where each process $Y_{i}$ ( i=1,2 ) describes the dynamic activity at the $i^{\text{th}}$ node. We remark that, in the context of bivariate time series analysis, when studying two dynamic processes (e.g., heart rate and blood pressure in physiology), researchers often look at how the oscillatory amplitudes in one signal relate to oscillatory amplitudes in another. Standard bivariate methods might use cross-correlation or coherence, which look at magnitude relationships without explicitly considering directional/phase aspects of the oscillation. In this context, it is worth mentioning the complex/rotary perspective, which usually refers to a way of representing oscillatory signals using complex numbers or rotary (circular) components, rather than just their real-valued time series (Sykulski et al., 2017). From a *complex* perspective, the signal Y(t) , with t the temporal variable, is represented in the complex plane, usually via the analytic signal $Y_{a}\left(t\right)$ obtained from its Hilbert transform $\hat{Y}\left(t\right)$ , i.e., $Y_{a}\left(t\right)=Y\left(t\right)+\text{j}\hat{Y}\left(t\right)$ , with $\text{j}=\sqrt{-1}$ . This captures both amplitude (magnitude) and phase (angle), giving a full oscillatory description of the signal; coupling metrics can be computed if treating each signal as complex separately, and take into account phase relationships, directionality, and rotations in phase space rather than just co-fluctuations in time (Rosenblum and Kurths, 1998). On the other hand, the *rotary* perspective looks at the overall bivariate oscillation a rotating vector (phasor) in the complex plane. Bivariate signals (e.g., $Y_{1}\left(t\right)$ and $Y_{2}\left(t\right)$ ) are often viewed together as a single complex signal, i.e., $Z\left(t\right)=Y_{1}\left(t\right)+\text{j}Y_{2}\left(t\right)$ . The signal Z(t) is decomposed into rotary components, namely, a positive-frequency (counter-clockwise) component and a negative-frequency (clockwise) component, to capture directionality of rotation, lead-lag relationships, and phase differences between the two signals that standard linear correlations would miss (Sykulski et al., 2017). Overall, these two perspectives can reveal directionality in oscillatory processes, e.g., one physiological variable leading or lagging another, forming loops in the phase plane. In spite of its usefulness and broad applicability, this area of research will not be deepened in the present work. Rather, we assume that the processes are *real-valued*, defined at discrete time ( $Y_{i}=\left\{Y_{i,n}\right\}$ ; e.g., are sampled versions of the continuous time processes $Y_{i}\left(t\right)$ , taken at the times $t_{n}=nT$ , with T the sampling period) and have zero mean ( $E\left[Y_{i,n}\right]=0$ , where E[⋅] is the statistical expectation operator).

### 2.2 Correlation-based measures in the time and frequency domain

In the time domain, the simplest way to identify symmetric statistical dependencies between signals is through *correlation*. The commonly used static approach disregards temporal dependencies and considers the zero-lag interaction between two random variables $V_{1}$ and $V_{2}$ whose observations are taken simultaneously (i.e., at the same time instant) from the two analysed time series. This interaction is typically quantified using the *Pearson correlation coefficient* (PCC) $\rho_{V_{1},V_{2}}$ , defined as the ratio between the covariance cov(⋅,⋅) of the two variables and the product of their standard deviations σ :

$$\rho_{V_{1},V_{2}}=\frac{cov\left(V_{1},V_{2}\right)}{\sigma_{V_{1}}\sigma_{V_{2}}},$$ (1)

where $cov\left(V_{1},V_{2}\right)=E\left[\left(V_{1}-m_{V_{1}}\right)\left(V_{2}-m_{V_{2}}\right)\right]$ ; $m_{V}=E\left[V\right]$ is the mean of V . Varying within the range [−1,1] , $\rho_{V_{1},V_{2}}$ quantifies the strength of the linear relationship between the variables, but cannot fully capture non-linear associations (Benesty et al., 2009).

To account for time-lagged dependencies, the time series are considered as realizations of two random processes $Y_{1}$ and $Y_{2}$ , from which the *cross-correlation function* (CCF) computed as a function of the time lag k as (Kay, 1988; Rodgers and Nicewander, 1988).

$$R_{Y_{1}Y_{2}}\left(k\right)=E\left[Y_{1,n}Y_{2,n-k}\right];$$ (2)

while the CCF in Equation 2 generally a function also of the time instant n , such dependence is omitted in Equation 2 under the hypothesis of WSS processes. The CCF captures how past values of $Y_{2}$ relate to current values of $Y_{1}$ in a linear signal processing framework, enabling the detection of lead-lag relationships. When estimated from data, this function helps identifying temporal dependencies and is often used as a precursor to more complex dynamic measures.

The two observed random processes can be studied in the frequency domain in terms of the power spectral density (PSD) matrix of the stationary bivariate random process $\text{Y}=\left\{Y_{1},Y_{2}\right\}$ . The PSD matrix, denoted as ${\text{P}}_{\text{Y}}\left(\bar{f}\right)$ , is a 2×2 matrix which contains the individual PSD of the process $Y_{i}$ , $P_{Y_{i}}\left(\bar{f}\right)$ , as $i^{\text{th}}$ diagonal element and the cross-PSD between the processes $Y_{i}$ and $Y_{j}$ , $P_{Y_{i}Y_{j}}\left(\bar{f}\right)$ , as off-diagonal elements in the position i−j ( i,j=1,2 ); ω∈[−π,π] is the normalized angular frequency ( $\omega =2\pi \frac{f}{f_{s}}=2\pi \bar{f}$ with $f\in \left[-\frac{f_{s}}{2},\frac{f_{s}}{2}\right]$ ; $\bar{f}$ normalized frequency, $f_{s}$ sampling frequency):

$${\text{P}}_{\text{Y}}\left(\bar{f}\right)=\left[\begin{array}{cc} P_{Y_{1}}\left(\bar{f}\right) & P_{Y_{1}Y_{2}}\left(\bar{f}\right)\\ P_{Y_{2}Y_{1}}\left(\bar{f}\right) & P_{Y_{2}}\left(\bar{f}\right) \end{array}\right].$$ (3)

The link between the time and frequency domain representations is provided by the Fourier Transform (FT) F{⋅} . Specifically, the PSD of the process $Y_{i},\left(i=1,2\right)$ , is defined as $P_{Y_{i}}\left(\bar{f}\right)=F\left\{R_{Y_{i}}\left(k\right)\right\}$ , with $R_{Y_{i}}\left(k\right)=E\left[Y_{i,n}Y_{i,n-k}\right]$ the autocorrelation function of $Y_{i}$ . Similarly, the cross-PSD between the two processes is defined as the FT of their CCF (Kay, 1988; Priestley, 1981).

$$P_{Y_{1}Y_{2}}\left(\bar{f}\right)=F\left\{R_{Y_{1}Y_{2}}\left(k\right)\right\}=\overset{\infty }{\underset{k=-\infty }{\sum }}R_{Y_{1}Y_{2}}\left(k\right)e^{-\text{j}2\pi \bar{f}k},$$ (4)

where $\text{j}=\sqrt{-1}$ ; the PSD of Equation 2 represents a fundamental tool in frequency domain analysis quantifying the extent to which two jointly stationary stochastic processes $Y_{1}$ and $Y_{2}$ co-vary as a function of frequency. By capturing both the magnitude and phase relationships of their frequency components, the cross-PSD provides insights into the presence, strength, and timing of linear interactions across different spectral bands, making it especially valuable in the analysis of oscillatory coupling in complex systems such as physiological (Faes et al., 2022; Sparacino et al., 2025a) or neural networks (Antonacci et al., 2021b). The cross-PSD is generally complex-valued, with its magnitude describing the co-oscillatory power at frequency $\bar{f}$ , and its phase indicating the relative timing or phase delay between the two signals at that frequency.

The *coherence* (Coh) between the two processes $Y_{1}$ and $Y_{2}$ can be defined as the ratio between the cross-spectrum $P_{Y_{1}Y_{2}}\left(\bar{f}\right)$ and the squared root of the product between the autospectra of $Y_{1}$ and $Y_{2}$

$$\Gamma_{Y_{1};Y_{2}}\left(\bar{f}\right)=\frac{P_{Y_{1}Y_{2}}\left(\bar{f}\right)}{\sqrt{P_{Y_{1}}\left(\bar{f}\right)P_{Y_{2}}\left(\bar{f}\right)}}.$$ (5)

Since this function is complex-valued, its squared modulus is commonly used to measure the strength of coupling in the frequency domain. Indeed, the magnitude squared coherence, $|\Gamma_{Y_{1};Y_{2}}\left(\bar{f}\right){|}^{2}$ , has a meaningful physical interpretation, since it measures the strength of the linear, non-directional coupled interactions between the processes $Y_{1}$ and $Y_{2}$ as a function of frequency. The magnitude squared coherence is a normalized measure of coupling, being 0 at a given frequency in case of uncoupling and 1 in case of deterministic linear dependence. Another popular, non-normalized spectral measure of coupling between two processes $Y_{1}$ and $Y_{2}$ is the logarithmic measure of *total dependence* (TD) defined by Geweke (1982) as

$$f_{Y_{1};Y_{2}}\left(\bar{f}\right)=\log\frac{P_{Y_{1}}\left(\bar{f}\right)P_{Y_{2}}\left(\bar{f}\right)}{\left|{\text{P}}_{\text{Y}}\left(\bar{f}\right)\right|};$$ (6)

exploiting Equation 5, it can be shown that the squared Coh is related to the logarithimc TD measure through the relation

$$f_{Y_{1};Y_{2}}\left(\bar{f}\right)=-\log\left(1-|\Gamma_{Y_{1};Y_{2}}\left(\bar{f}\right){|}^{2}\right).$$ (7)

In practice, estimation of the PSD from finite-length time series can be achieved through both parametric (Section 2.4) and non-parametric (Section 4) methods, each with its own strengths and weaknesses (Kay, 1988; Pinna et al., 1996; Zhao and Gui, 2019). The choice of the method depends on the characteristics of the signal, the available data, and the specific requirements of the analysis, being often a trade-off between frequency resolution, variance reduction, and computational complexity.

### 2.3 Information-theoretic measures of coupling and causality

In this section, we present the well-known information-theoretic measures used for analyzing undirected and directed interactions in bivariate systems. Each of the measures is described with detail; the characterization of their mathematical relationships allows to highlight how they capture distinct aspects of statistical dependence.

#### 2.3.1 Mutual information and mutual information rate

The most popular measure of coupling derived in the frame of information theoryis the *mutual information* (MI). The MI is a symmetric measure quantifying the amount of information shared by two random variables $V_{1}$ and $V_{2}$ , intended as the uncertainty about one of the variables that is resolved by knowing the other. Formally, the MI is defined as (Shannon, 1948; Cover, 1999):

$$I\left(V_{1};V_{2}\right)=E\left[\log\frac{p_{V_{1},V_{2}}\left(v_{1},v_{2}\right)}{p_{V_{1}}\left(v_{1}\right)p_{V_{2}}\left(v_{2}\right)}\right],$$ (8)

where p(⋅,⋅) and p(⋅) represent the joint and marginal probability distributions, respectively. The MI defined in Equation 8 can be equivalently expressed in terms of Shannon entropies as:

$$I\left(V_{1};V_{2}\right)=H\left(V_{1}\right)+H\left(V_{2}\right)-H\left(V_{1},V_{2}\right),$$ (9)

where H(⋅) denotes entropy and H(⋅,⋅) denotes joint entropy (Shannon, 1948; Cover, 1999). The MI is intimately linked to the PCC (Equation 1) when the two random variables $V_{1}$ and $V_{2}$ have a joint Gaussian distribution; if this is the case, the MI between $V_{1}$ and $V_{2}$ can be expressed analytically as (Jwo et al., 2023):

$$I\left(V_{1};V_{2}\right)=-\frac{1}{2}\log\left(1-\rho_{V_{1},V_{2}}^{2}\right);$$ (10)

Equation 10 establishes a direct and quantitative link between linear correlation and information-theoretic dependence. It is worth stressing that the MI, like the PCC, measures the static interaction between two random variables.

The *mutual information rate* (MIR) generalizes the MI between random variables by quantifying the dynamic coupling between the two stochastic processes $Y_{1}$ and $Y_{2}$ . Specifically, the MIR measures the amount of information shared per unit of time between the processes, and is defined as (Duncan, 1970; Geweke, 1982; Sparacino et al., 2025a):

$$I_{Y_{1};Y_{2}}=\underset{q\to \infty }{\lim}\frac{1}{q}I\left(Y_{1,n}^{q};Y_{2,n}^{q}\right)=I\left(Y_{1,n},Y_{1,n}^{-};Y_{2,n},Y_{2,n}^{-}\right)-I\left(Y_{1,n}^{-},Y_{2,n}^{-}\right),$$ (11)

where I(⋅;⋅) denotes MI. In essence, the MIR quantifies the overall coupling between the two processes elaborating the MI between all their constituent variables; note that the MI computed between a sequence of random variables composing two processes needs to be divided by the number of these variables to yield a non-diverging quantity. As the MI, the MIR is a symmetric measure (i.e., $I_{Y_{1};Y_{2}}=I_{Y_{2};Y_{1}}$ ) and is widely used to characterize bivariate dynamic interactions across various scientific domains [see, e.g., Baptista and Kurths (2008); Pinto et al. (2025d); Sparacino et al. (2025a)]. The MIR can be straightforwardly decomposed into information-theoretic quantities that offer valuable insights into the complex dynamics of the individual components within a bivariate system:

$$I_{Y_{1};Y_{2}}=H_{Y_{1}}+H_{Y_{2}}-H_{Y_{1},Y_{2}},$$ (12)

where $H_{Y_{1}}=H\left(Y_{1,n}|Y_{1,n}^{-}\right)$ and $H_{Y_{2}}=H\left(Y_{2,n}|Y_{2,n}^{-}\right)$ denote the entropy rates of $Y_{1}$ and $Y_{2}$ , respectively, and $H_{Y_{1},Y_{2}}=H\left(Y_{1,n},Y_{2,n}|Y_{1,n}^{-},Y_{2,n}^{-}\right)$ their joint entropy rate (with H(⋅|⋅) denoting conditional entropy) (Cover, 1999; Chicharro, 2011). Note that the MIR expressed as in Equation 12 is formally equivalent to the MI expressed as in Equation 9, with the use of entropy rate terms in place of standard entropy terms. Another important meaningful decomposition of the MIR is derived in the next subsection.

We anticipate that, in the case of stationary Gaussian processes, the MIR is closely connected to a well-known time-domain measure of non-directional dynamic coupling related to the concept of TD developed in the context of linear regression models (Geweke, 1982); the relevant derivations will be provided in Section 2.

#### 2.3.2 Causal and instantaneous information transfer

Inferring directional interactions between time series is a fundamental task in the analysis of dynamical systems. Generally, the two random processes representing the dynamic activity of the units interact in a closed-loop manner, i.e., through bidirectional and reciprocal influences which allow to identify asymmetrical driver-response patterns (Porta and Faes, 2015).

In the field of information theory, a widely used measure for assessing such interactions is *transfer entropy* (TE), formally defined as (Schreiber, 2000):

$$\begin{array}{cc} T_{Y_{1}\to Y_{2}} & =I\left(Y_{2,n};Y_{1,n}^{-}|Y_{2,n}^{-}\right)=H\left(Y_{2,n}|Y_{2,n}^{-}\right)-H\left(Y_{2,n}|Y_{1,n}^{-},Y_{2,n}^{-}\right)\\ & =H\left(Y_{2,n},Y_{2,n}^{-}\right)-H\left(Y_{2,n}^{-}\right)-H\left(Y_{2,n},Y_{1,n}^{-},Y_{2,n}^{-}\right)+H\left(Y_{1,n}^{-},Y_{2,n}^{-}\right), \end{array}$$ (13)

where I(⋅;⋅∣⋅) denotes conditional MI; herein, $Y_{1}$ is assumed as the *driver* process, while $Y_{2}$ as the *target* process.

Remarkably, the MIR can be formulated comparing the sum of the 2 TEs from $Y_{1}$ to $Y_{2}$ and from $Y_{2}$ to $Y_{1}$ (Barà et al., 2023):

$$I_{Y_{1};Y_{2}}=T_{Y_{1}\to Y_{2}}+T_{Y_{2}\to Y_{1}}+I_{Y_{1}\cdot Y_{2}},$$ (14)

where $T_{Y_{1}\to Y_{2}}$ and $T_{Y_{2}\to Y_{1}}$ represent the directional information flow from $Y_{1}$ to $Y_{2}$ and from $Y_{2}$ to $Y_{1}$ , respectively. The term $I_{Y_{1}\cdot Y_{2}}$ , commonly referred to as *instantaneous transfer* (IT) (Chicharro, 2011), captures the instantaneous, bidirectional exchange of information between $Y_{1}$ and $Y_{2}$ and is defined as:

$$\begin{array}{cc} I_{Y_{1}\cdot Y_{2}} & =I\left(Y_{1,n};Y_{2,n}|Y_{1,n}^{-},Y_{2,n}^{-}\right)\\ & =H\left(Y_{1,n}|Y_{1,n}^{-},Y_{2,n}^{-}\right)-H\left(Y_{1,n}|Y_{2,n},Y_{1,n}^{-},Y_{2,n}^{-}\right). \end{array}$$ (15)

We remark that the TE is a well-known measure of directional information transfer related to the concept of Granger causality (GC) originally developed by Wiener (Wiener, 1956) and then made operative by Granger in the context of linear regression models (Granger, 1969), while the IT is a symmetric measure related to the concept of instantaneous causality (IC) (Chicharro, 2011).

## 3 Implementation through linear autoregressive models

This section presents the definition and practical implementation of the time, spectral and information-theoretic measures of directed and undirected coupling obtained through linear regression methods making use of univariate and bivariate autoregressive (AR) models. Linear AR models are ubiquitously used to assess dynamics and interactions in time series data, especially in the field of Network Physiology (Bressler and Seth, 2011; Seth et al., 2015; Porta and Faes, 2015; Faes et al., 2016b; Sparacino et al., 2024b; Vakitbilir et al., 2025). Here, we begin discussing three issues that have theoretical relevance and practical implications in the use of AR models for the computation of interaction measures.

The first observation is that fitting linear AR models on time series assumes linearity in the modelled interactions, but not in the processes to be analysed: indeed, while the model assumes linearity in its structure, this does not necessarily imply that the underlying time series must be linear (Barnett and Seth, 2023). Indeed, Wold’s decomposition theorem (Wold, 1938) guarantees that any stationary process can be decomposed into a linear model, although this model may be of infinite order and thus providing a non-parsimonious representation of an underlying nonlinear process (Hannan, 1979). Therefore, linear AR models may in principle be able to describe also the dynamics of processes with nonlinear generating mechanisms.

Another relevant observation is that, while linear AR models can be used to describe time series with any probability distribution, when the underlying processes are jointly Gaussian distributed the measures derived in the time domain (Barrett et al., 2010; Faes et al., 2016b) and in the spectral domain (Chicharro, 2011; Faes et al., 2021; Antonacci et al., 2021b; Sparacino et al., 2025a) have a striking information-theoretic interpretation. In fact, the parametric implementation exploits the knowledge that linear regression models capture all of the entropy differences relevant to the various information measures when the observed processes have a joint Gaussian distribution (Barrett et al., 2010; Faes et al., 2016b).

The third point regards the fact that linear AR models typically limit to past values only the possible influences of one process to another, thereby excluding instantaneous effects (i.e., effects occurring within the same lag) from the model structure (Lütkepohl, 2005). The consequence of this is that the model residuals (prediction errors) are correlated whenever instantaneous effects are present between the analysed time series. On the other hand, the absence of instantaneous effects, typically denoted as *strict causality* of the process (Korhonen et al., 1996; Baselli et al., 1997) implies that the covariance matrix of the residuals is diagonal. While strict causality is often assumed in the computation of causality measures, the presence of instantaneous effects has an impact on the derived measures. In the following subsections we will discuss such an impact and mention how bivariate measures coupling and causality measures can be corrected to account for instantaneous effects.

### 3.1 Formulation of linear parametric models of bivariate time series

The linear formulation leading to compute coupling measures requires identification of a bivariate AR model composed by two so-called full *auto- and cross-regressive* (ARX) models, from which restricted *autoregressive* (AR) models are derived to compute causality measures. Full ARX models feature two model equations, where the present states of the two processes are written as linear combinations of the past states of both processes weighted by a set of model coefficients plus the residuals. Assuming that Y is the generic vector process comprising the two scalar processes $\left\{Y_{1},Y_{2}\right\}$ , the following ARX model can be identified (Lütkepohl, 2005):

$$Y_{1,n}=\overset{p}{\underset{k=1}{\sum }}a_{Y_{1}Y_{1},k}Y_{1,n-k}+a_{Y_{1}Y_{2},k}Y_{2,n-k}+U_{Y_{1},n},$$ (16a)

$$Y_{2,n}=\overset{p}{\underset{k=1}{\sum }}a_{Y_{2}Y_{1},k}Y_{1,n-k}+a_{Y_{2}Y_{2},k}Y_{2,n-k}+U_{Y_{2},n},$$ (16b)

where ${\text{Y}}_{n}={\left[Y_{1,n},Y_{2,n}\right]}^{⊺}$ is the 2-dimensional vector collecting the present state of the two processes, $A_{\text{Y},k}=\left[\begin{array}{cc} a_{Y_{1}Y_{1},k} & a_{Y_{1}Y_{2},k}\\ a_{Y_{2}Y_{1},k} & a_{Y_{2}Y_{2},k} \end{array}\right]$ is the 2×2 matrix of the model coefficients relating the present with the past of the two processes at lag k , and ${\text{U}}_{\text{Y},n}={\left[U_{Y_{1},n},U_{Y_{2},n}\right]}^{⊺}$ a vector of 2 zero-mean white noises with 2×2 positive definite covariance matrix $\Sigma_{{\text{U}}_{\text{Y}}}=\left[\begin{array}{cc} \sigma_{U_{Y_{1}}}^{2} & \sigma_{U_{Y_{1}Y_{2}}}^{2}\\ \sigma_{U_{Y_{2}Y_{1}}}^{2} & \sigma_{U_{Y_{2}}}^{2} \end{array}\right]$ . Note that $\sigma_{U_{Y_{1}Y_{2}}}^{2}=\sigma_{U_{Y_{2}Y_{1}}}^{2}=0$ in the case of strict causality, implying the absence of instantaneous interactions. The process ${\text{Y}}_{n}$ has a 2×2 covariance matrix $\Sigma_{\text{Y}}=E\left[{\text{Y}}_{n}{\text{Y}}_{n}^{⊺}\right]$ , where the diagonal elements represent the variances of the scalar processes $\left\{Y_{1},Y_{2}\right\}$ , i.e., $\sigma_{Y_{1}}^{2}$ , $\sigma_{Y_{2}}^{2}$ .

#### 3.1.1 Model identification

The identification procedure of the ARX model in Equations 16a, b is typically performed by means of estimation methods based on minimizing the prediction error, i.e., the difference between actual and predicted data (Kay, 1988; Lütkepohl, 2005). While several approaches have been proposed throughout the years (Schlögl, 2006; Antonacci et al., 2020), the most common estimator is the multivariate version of the *ordinary least-squares* (OLS) method (Lütkepohl, 2005). Briefly, defining the past history of Y truncated at p lags as the 2p -dimensional vector ${\text{Y}}_{n}^{p}={\left[{\text{Y}}_{n-1}^{⊺},\ldots ,{\text{Y}}_{n-p}^{⊺}\right]}^{⊺}$ and considering L consecutive time steps, a compact representation of Equations 16a,b can be defined as $\text{Y}=A_{\text{Y}}{\text{Y}}^{p}+{\text{U}}_{\text{Y}}$ , where $A_{\text{Y}}=\left[A_{\text{Y},1},\ldots ,A_{\text{Y},p}\right]$ is the 2×2p matrix of unknown coefficients, $\text{Y}=\left[{\text{Y}}_{p+1},\ldots ,{\text{Y}}_{L}\right]$ and ${\text{U}}_{\text{Y}}=\left[{\text{U}}_{\text{Y},p+1},\ldots ,{\text{U}}_{\text{Y},L}\right]$ are 2×(L−p) matrices, and ${\text{Y}}^{p}=\left[{\text{Y}}_{p+1}^{p},\ldots ,{\text{Y}}_{L}^{p}\right]$ is a 2p×(L−p) matrix collecting the regressors. The method estimates the coefficient matrices through the OLS formula, ${\hat{A}}_{\text{Y}}=\text{Y}{\left({\text{Y}}^{p}\right)}^{⊺}{\left[{\text{Y}}^{p}{\left({\text{Y}}^{p}\right)}^{⊺}\right]}^{-1}$ . The innovation process is estimated as the residual time series ${\hat{\text{U}}}_{\text{Y}}=\text{Y}-{\hat{A}}_{\text{Y}}{\text{Y}}^{p}$ , whose covariance matrix ${\hat{\Sigma }}_{{\text{U}}_{\text{Y}}}$ is an estimate of $\Sigma_{{\text{U}}_{\text{Y}}}$ . After identification, the model in Equations 16a,b can be analyzed in the frequency domain.

As regards the selection of the model order p , several criteria exist for its determination [see, e.g., Lütkepohl (2005); Karimi (2011)]. One commonly used approach is to set the order according to the *Akaike Information Criterion* (AIC) (Akaike, 1974), or the *Bayesian Information Criterion* (BIC) (Schwarz, 1978). The primary difference between AIC and BIC lies in how they penalize model complexity and their underlying theoretical foundations. AIC is based on information theory and aims to minimize the information lost when using a model to approximate the true process. It focuses on predictive accuracy and is more likely to select models that perform well for future data. The penalty for the number of parameters is more moderate with AIC, which indeed balances goodness of fit with model complexity but places less emphasis on the number of parameters. On the other hand, BIC approximates the posterior probability of a model given the data, and seeks the model that is most likely to be the true one, based on the given data, and is more concerned with identifying the correct model. Penalty grows with the sample size using BIC, which heavily penalizes models with more parameters, especially in larger datasets. Practically, it is crucial to find the right balance between excessively low orders, which might lead to an inadequate description of crucial oscillatory information in the vector process, and overly high orders, which could result in overfitting, with the outcome that the model captures not only the desired information but also includes noise. Additional guidelines on model order selection include 1) utilizing multiple selection criteria and checking for consistency; if they converge on similar orders, confidence in the choice increases. 2) Fitting models at nearby orders ( ±1−2 ) could help in assessing stability, since large changes in spectral measures or AR coefficients suggest sensitivity to model order. 3) Finally, visually inspecting the AR-derived PSD may be helpful, especially in the case of physiological variables whose spectral behaviour is generally well-known: overly smooth spectra may indicate too low orders, while unrealistic peaks or high-frequency noise suggest overfitting (Pernice et al., 2022a). As regards possible non-stationarity in the data, cutting large non-stationary physiological signals to identify shorter stationary segments may be a practical solution; this reduces sensitivity to model order, as the local dynamics can be captured with lower orders. As an example, for short-window heart rate variability series (on the order of ∼300 beats), parametric modelling via AR methods has been used for complexity and spectral assessment [e.g., Porta et al. (2002b), Porta et al. (2017)]. In keeping with the precedent of moderate orders in such short windows, model orders in the range 5−8 are a good compromise between spectral resolution and robustness (see Section 5.1).

#### 3.1.2 Restricted AR model

While the ARX model in Equations 16a,b provides a global representation of the overall bivariate process, to describe the linear interactions relevant to, e.g., the target process, we need to define a restricted AR model involving only $Y_{2}$ . To implement this concept, the present state of the target, $Y_{2,n}$ , is described first from the past of $Y_{2}$ only through the *restricted AR model*

$$Y_{2,n}=\overset{\infty }{\underset{k=1}{\sum }}b_{Y_{2}Y_{2},k}Y_{2,n-k}+W_{Y_{2},n},$$ (17)

where $b_{Y_{2}Y_{2},k}$ are AR coefficients and $W_{Y_{2}}$ is a white noise process with variance $\lambda_{W_{Y_{2}}}^{2}$ .

An issue with great practical relevance is that the order of the restricted model in Equation 17 is typically infinite and thus very difficult to identify from finite-length time series. The approach followed to face this issue in the context of causality analysis is essentially based on truncating the order of the restricted model to p , and estimating its parameters from the relevant subset of the original data. Though simple, this approach exposes to a trade-off between bias and variance of the estimates that prevents reliable model identification in most cases (Stokes and Purdon, 2017; Faes et al., 2017b). To solve this issue, methods which essentially extract the parameters of the restricted model from those of the full model have been proposed, i.e., methods based on *state-space (SS) models* (Faes et al., 2017b; Barnett et al., 2018) and on the resolution of the *Yule-Walker (YW) equations* (Barnett and Seth, 2014; Faes et al., 2015b; 2016b; Sparacino et al., 2024a). In the following, the two methods will be thoroughly described.

### 3.2 Identification of restricted models

#### 3.2.1 State-space models

The method based on SS models can be applied to the bivariate AR model in Equations 16a,b to derive the parameters of the two corresponding restricted AR models of $Y_{1},Y_{2}$ in the form of Equation 17, i.e., $\left\{b_{Y_{1}Y_{1}},\lambda_{W_{Y_{1}}}^{2}\right\}$ and $\left\{b_{Y_{2}Y_{2}},\lambda_{W_{Y_{2}}}^{2}\right\}$ , respectively (Barnett and Seth, 2015). This class of models is the most appropriate to use because it is closed under the formation of restricted models: in fact, any restricted process obtained from a vector AR process is actually an AR process with a moving average component, or equivalently a finite-order SS process (Barnett et al., 2018). Therefore, using SS models allows to identify restricted models from the parameters of the original vector AR model estimated with a single regression, thus guaranteeing high computational reliability. We exploit SS modelling to describe the original process Y obeying the bivariate AR representation in Equations 16a,b using the SS model.

$${\text{S}}_{n+1}=A{\text{S}}_{n}+K{\text{U}}_{\text{Y},n},$$ (18a)

$${\text{Y}}_{n}=C{\text{S}}_{n}+{\text{U}}_{\text{Y},n},$$ (18b)

where ${\text{S}}_{n}={\left[{\text{Y}}_{n-1}^{⊺},\ldots ,{\text{Y}}_{n-p}^{⊺}\right]}^{⊺}$ is the 2p -dimensional state process and the SS parameters ( A,C,K,V ) are given by the matrices $C=\left[A_{\text{Y},1},\ldots ,A_{\text{Y},p}\right]$ , $K={\left[I_{2}0_{2\times 2\left(p-1\right)}\right]}^{⊺}$ , $A=\left[C;I_{2\left(p-1\right)}0_{2\left(p-1\right)\times 2}\right]$ , and $V=E\left[{\text{U}}_{\text{Y},n}{{\text{U}}_{\text{Y},n}}^{⊺}\right]=\Sigma_{{\text{U}}_{\text{Y}}}$ ( I and 0 are the identity and null matrices, respectively). Then, to represent the scalar process $Y_{2}$ , we replace Equation 17 with a restricted SS model with state equation as in Equation 18a and observation equation $Y_{2,n}=C^{\left(2,:\right)}{\text{S}}_{n}+W_{Y_{2},n}$ . The parameters of the model are ( $A,C^{\left(2,:\right)},KVK^{⊺},V^{\left(2,2\right)},KV^{\left(:,2\right)}$ ), where the superscripts denote selection of the rows and/or columns with indices 2 in a matrix. To exploit the restricted SS model for the linear causality analysis of $Y_{2}$ it is necessary to lead its form back to that of Equations 18a,b, which reads (Barnett and Seth, 2015)

$${\text{S}}_{n+1}=\tilde{A}{\text{S}}_{n}+\tilde{K}W_{Y_{2},n},$$ (19a)

$$Y_{2,n}=\tilde{C}{\text{S}}_{n}+W_{Y_{2},n}.$$ (19b)

The parameters of the restricted model in Equations 19a,b are ( $\tilde{A},\tilde{C},\tilde{K},\tilde{V}$ ), of dimension 2p×2p,1×2p,2p×1,1×1 , and can be derived directly from the parameters $A_{\text{Y},k}$ and $\Sigma_{{\text{U}}_{\text{Y}}}$ of the original full ARX model in Equations 16a,b (Barnett and Seth, 2015): while the state and observation matrices are easily determined as $\tilde{A}=A$ and $\tilde{C}=C^{\left(2,:\right)}$ , the gain $\tilde{K}$ and the restricted innovation covariance $\tilde{V}=\lambda_{W_{Y_{2}}}^{2}$ must be obtained by solving a discrete algebraic Riccati equation (DARE) (see Barnett and Seth (2015); Faes et al. (2017a) for detailed derivations). After identification, the model in Equations 19a,b can be analyzed in the frequency domain to study spectral interactions relevant to the process $Y_{2}$ .

#### 3.2.2 Resolution of the Yule-Walker equations

The issue related to the formation of AR restricted models from the ARX model in Equations 16a,b can be overcome also by solving the YW equations. The restricted model coefficients, $b_{Y_{2}Y_{2},k}$ , and the variance of the residuals, $\lambda_{W_{Y_{2}}}^{2}$ , appearing in Equation 17, can be identified starting from the covariance and cross-covariance matrices between the present and past variables of the two scalar processes $Y_{1}$ and $Y_{2}$ . Using these matrices allows to identify restricted models from the parameters of the original ARX model estimated with a single regression up to an arbitrarily large order q , thus guaranteeing computational reliability (Barnett and Seth, 2014; Faes et al., 2015b; 2016b; Mijatovic et al., 2025). For jointly Gaussian processes, these matrices contain as scalar elements the covariance between two time-lagged variables taken from the processes $Y_{1}$ and $Y_{2}$ , which in turn appear as elements of the 2×2 autocovariance of the whole observed 2-dimensional process ${\text{Y}}_{n}={\left[Y_{1,n}Y_{2,n}\right]}^{⊺}$ , defined at each lag k≥0 as $\Gamma_{k}=E\left[{\text{Y}}_{n}{\text{Y}}_{n-k}^{⊺}\right]$ . The procedure exploits the possibility to compute $\Gamma_{k}$ from the parameters of the ARX formulation of the process ${\text{Y}}_{n}$ via the well-known YW equations:

$$\Gamma_{k}=\overset{p}{\underset{l=1}{\sum }}{\text{A}}_{\text{Y},l}\Gamma_{k-l}+\delta_{k0}\Sigma_{{\text{U}}_{\text{Y}}},$$ (20)

where $\delta_{k0}$ is the Kronecker delta function. In order to solve Equation 20 for $\Gamma_{k}$ , with k=0,…,p−1 , we first express the ARX model in Equations 16a,b in compact form as $\psi_{n}=\text{A}\psi_{n-1}+{\text{E}}_{n}$ , where:

$$\begin{array}{cc} & \psi_{n}={\left[{\text{Y}}_{n}^{⊺}{\text{Y}}_{n-1}^{⊺},\ldots ,{\text{Y}}_{n-p+1}^{⊺}\right]}^{⊺};\\ & \text{A}=\left[\begin{array}{cccc} {\text{A}}_{\text{Y},1} & \ldots & {\text{A}}_{\text{Y},p-1} & {\text{A}}_{\text{Y},p}\\ \text{I} & \ldots & 0 & 0\\ \vdots & \ddots & \vdots & \vdots \\ 0 & \ldots & \text{I} & 0 \end{array}\right];\\ & {\text{E}}_{n}={\left[{{\text{U}}_{\text{Y},n}}^{⊺}0_{1\times 2\left(p-1\right)}\right]}^{⊺}. \end{array}$$ (21)

From Equation 21, the 2p×2p covariance matrix of $\psi_{n}$ , which is defined as $\Psi =E\left[\psi_{n}\psi_{n}^{⊺}\right]$ and has the form

$$\Psi =\left[\begin{array}{cccc} \Gamma_{0} & \Gamma_{1} & \ldots & \Gamma_{p-1}\\ \Gamma_{1}^{⊺} & \Gamma_{0} & \ldots & \Gamma_{p-2}\\ \vdots & \vdots & \ddots & \vdots \\ \Gamma_{p-1}^{⊺} & \Gamma_{p-2}^{⊺} & \ldots & \Gamma_{0} \end{array}\right],$$ (22)

can be expressed as $\Psi =\text{A}\Psi {\text{A}}^{⊺}+\Xi$ where $\Xi =E\left[{\text{E}}_{n}{\text{E}}_{n}^{⊺}\right]$ is the 2p×2p covariance of ${\text{E}}_{n}$ . This last equation is a discrete-time Lyapunov equation, which can be solved for Ψ appearing in Equation 22 to yield the autocovariance matrices $\Gamma_{0},\ldots ,\Gamma_{p-1}$ (Faes et al., 2015b; Mijatovic et al., 2025). Note that $\Gamma_{0}\equiv \Sigma_{\text{Y}}$ . Finally, the autocovariance can be calculated recursively for any lag k≥p by repeatedly applying YW equations in Equations 16a,b up to the desired lag q , starting from the parameters of the ARX representation in Equations 16a,b of the observed Gaussian vector process Y .

Then, once the covariance matrices $\Gamma_{0},\ldots ,\Gamma_{q}$ are derived, they need to be pruned to retain only those elements which relate to the desired restricted model. Here, we review the procedure which allows to get the restricted AR parameters $\left\{b_{Y_{2}Y_{2},k},\lambda_{W_{Y_{2}}}^{2}\right\}$ . The AR model in Equation 17 can be written in compact form as $Y_{2,n}={\text{B}}_{Y_{2}Y_{2}}Y_{2,n}^{q}+W_{Y_{2},n}$ , where ${\text{B}}_{Y_{2}Y_{2}}=\left[b_{Y_{2}Y_{2},1},\ldots ,b_{Y_{2}Y_{2},q}\right]$ is the vector collecting all coefficients up to lag q . From this representation, taking the expectation $E\left[Y_{2,n}Y_{2,n}^{q^{⊺}}\right]$ and solving for ${\text{B}}_{Y_{2}Y_{2}}$ yields:

$${\text{B}}_{Y_{2}Y_{2}}=\Sigma_{Y_{2,n},Y_{2,n}^{q}}\cdot \Sigma_{Y_{2,n}^{q}}^{-1},$$ (23)

where in Equation 23 the covariance $\Sigma_{Y_{2,n}^{q}}=E\left[Y_{2,n}^{q}Y_{2,n}^{q^{⊺}}\right]$ is the q×q autocovariance matrix of $Y_{2,n}^{q}$ , while $\Sigma_{Y_{2,n}Y_{2,n}^{q}}=E\left[Y_{2,n}Y_{2,n}^{q^{⊺}}\right]$ is the 1×q cross-covariance matrix of $Y_{2,n}$ and $Y_{2,n}^{q}$ . The matrices $\Sigma_{Y_{2,n}^{q}}$ and $\Sigma_{Y_{2,n}Y_{2,n}^{q}}$ are extracted from $\Gamma_{k}$ . Then, the variance of the AR residuals $\lambda_{W_{Y_{2}}}^{2}$ in Equation 17 is computed as (Barnett et al., 2009):

$$\lambda_{W_{Y_{2}}}^{2}=\sigma_{Y_{2}}^{2}-\Sigma_{Y_{2,n},Y_{2,n}^{q}}\cdot \Sigma_{Y_{2,n}^{p}}^{-⊺}\cdot \Sigma_{Y_{2,n},Y_{2,n}^{q}}^{⊺}.$$ (24)

To summarize, the above-described procedure is based first on computing the autocovariance sequence of the bivariate process Y from its parameters ( ${\text{A}}_{\text{Y},l}$ , with l=1,…,p , and $\Sigma_{{\text{U}}_{\text{Y}}}$ ), which are previously identified through the vector OLS approach, and then on rearranging the elements of the autocovariance matrices for building the auto- and cross-covariances to be used in the computation, according to Equation 23, 24, of the AR parameters $\left\{b_{Y_{2}Y_{2},k},\lambda_{W_{Y_{2}}}^{2}\right\}$ appearing in Equation 17. The identification of the restricted model in Equation 17 can be represented in the frequency domain to study spectral patterns of causality. The same procedure applies to the AR parameters $\left\{b_{Y_{1}Y_{1},k},\lambda_{W_{Y_{1}}}^{2}\right\}$ if $Y_{1}$ is assumed as the target process.

Contrary to the closed-form SS modeling approach presented in the previous subsection, the procedure described here is approximate because it retains the AR structure which has infinite order for the restricted model. The parameter determining the accuracy of the procedure is the number of lags used to truncate the past history of the process: considering the past up to lag q corresponds to calculating the autocovariance of the process in Equations 16a,b up to the matrix $\Gamma_{q}$ . Given that the autocovariance of a stable vector AR process decays exponentially with the lag, with a rate of decay depending on the modulus of the largest eigenvalue of A , ρ(A) , it has been suggested to compute the autocovariance up to a lag q such that ρ(A) is smaller than a predefined numerical tolerance (Barnett and Seth, 2014). It has been found that computation of very long autocovariance sequences is not necessary for the purpose of evaluating information dynamics, because all measures stabilize to constant values already for small lags (typically q=10 ) even for reasonably high values of ρ(A) (Faes et al., 2013b; 2015a; b, 2016b; Mijatovic et al., 2025). In fact, the procedure described above yields results similar to the method of SS models with q sufficiently large (Antonacci et al., 2021a).

### 3.3 Time domain measures for linear processes

The parametric representation of the analysed bivariate process allows to derive measures of coupling and causality which are widely used for the description of symmetric and directed interactions in the time domain (Geweke, 1982; Bressler and Seth, 2011; Porta and Faes, 2015). These measures are obtained from the variances of the two analysed processes and of the prediction errors of the full and restricted models of Equations 16a,b, Equations 17. Specifically, a time-domain measure of the TD between the two random processes $Y_{1}$ and $Y_{2}$ is obtained comparing the generalized covariance of the vector noise ${\text{U}}_{\text{Y},n}$ of the full bivariate model of Equations 16a,b with the individual noise variances $W_{Y_{1}}$ and $W_{Y_{2}}$ of two separate restricted models in the form of Equation 17 as follows (Geweke, 1982; Pernice et al., 2022b)

$$F_{Y_{1};Y_{2}}=\log\frac{\lambda_{W_{Y_{1}}}^{2}\lambda_{W_{Y_{2}}}^{2}}{\left|\Sigma_{{\text{U}}_{\text{Y}}}\right|}.$$ (25)

The TD measure (Equation 25) is zero in the absence of any interaction between the two processes, resulting from a full equivalence between the bivariate AR description of $\left\{Y_{1},Y_{2}\right\}$ and the two individual AR descriptions of $Y_{1}$ and $Y_{2}$ ; if this occurs, the two restricted models have the same error variance of the full model ( $\lambda_{W_{Y_{1}}}^{2}=\sigma_{U_{Y_{1}}}^{2}$ , $\lambda_{W_{Y_{2}}}^{2}=\sigma_{U_{Y_{2}}}^{2}$ ) and the two errors of the full model are uncorrelated ( $\sigma_{U_{Y_{1}Y_{2}}}^{2}=0$ ). On the contrary, $F_{Y_{1};Y_{2}}$ grows with the strength of the causal influence from $Y_{1}$ to $Y_{2}$ (yielding $\sigma_{U_{Y_{2}}}^{2}<\lambda_{W_{Y_{2}}}^{2}$ ), with the strength of the causal influence from $Y_{2}$ to $Y_{1}$ (yielding $\sigma_{U_{Y_{1}}}^{2}<\lambda_{W_{Y_{1}}}^{2}$ ), or with the strength of the instantaneous effects (yielding $\sigma_{U_{Y_{1}Y_{2}}}^{2}=\sigma_{U_{Y_{2}Y_{1}}}^{2}>0$ ). These three components of the coupling can be isolated by decomposing the TD measure as (Geweke, 1982):

$$F_{Y_{1};Y_{2}}=F_{Y_{1}\to Y_{2}}+F_{Y_{2}\to Y_{1}}+F_{Y_{1}\cdot Y_{2}}.$$ (26)

Equation 26 evidences evidences the two measures of Granger causality (GC) from $Y_{1}$ to $Y_{2}$ and from $Y_{2}$ to $Y_{1}$ , $F_{Y_{1}\to Y_{2}}$ and $F_{Y_{2}\to Y_{1}}$ , and the measure of instantaneous causality (IC), $F_{Y_{1}\cdot Y_{2}}$ . The GC measures quantify the directed interactions between the processes according to the principle of Granger causality (Granger, 1969), whereby the improvement in predictability of the present state of a process brought by the knowledge of the past states of the other process can be quantified comparing the prediction error variances of the full and the restricted AR models of Equations 16a,b, Equations 17; specifically, GC from $Y_{1}$ to $Y_{2}$ is quantified as:

$$F_{Y_{1}\to Y_{2}}=\log\frac{\lambda_{W_{Y_{2}}}^{2}}{\sigma_{U_{Y_{2}}}^{2}},$$ (27)

and GC from $Y_{2}$ to $Y_{1}$ is computed analogously exchanging the role of the two processes. Finally, the IC measure quantifies the zero-lag correlation between the two processes, which is reflected by the off-diagonal elements of the covariance ${\text{U}}_{\text{Y},n}$ of the errors of the bivariate AR model in Equation 16:

$$F_{Y_{1}\cdot Y_{2}}=\log\frac{\sigma_{U_{Y_{1}}}^{2}\sigma_{U_{Y_{2}}}^{2}}{\left|\Sigma_{{\text{U}}_{\text{Y}}}\right|};$$ (28)

in the case of strict causality ( $\sigma_{U_{Y_{1}Y_{2}}}^{2}=\sigma_{U_{Y_{2}Y_{1}}}^{2}=0$ ) the IC measure of Equation 28 vanishes, denoting the absence of instantaneous effects.

### 3.4 Spectral measures for linear processes

In this section we present the tools whereby the linear parametric description of time series is widely exploited to describe coupling and causality in the frequency domain in a range of applicative fields, primarily including network neuroscience and network physiology (Schulz et al., 2013; Porta and Faes, 2015; Santiago-Fuentes et al., 2022; Sorelli et al., 2022; Jahani et al., 2025). To achieve a parametric estimation of the PSD matrix of the process (Equation 3), the ARX model in Equation 16 can be represented in the Z-domain through its Z-transforms yielding $\text{Y}\left(z\right)=H\left(z\right){\text{U}}_{\text{Y}}\left(z\right)$ , where $H\left(z\right)={\left[\text{I}-\sum_{k=1}^{p}A_{\text{Y},k}z^{-k}\right]}^{-1}={\bar{A}}_{\text{Y}}{\left(z\right)}^{-1}$ is the 2×2 transfer matrix (TF) matrix modelling the relationships between the input ${\text{U}}_{\text{Y}}\left(z\right)$ and the output Y(z) . Computing H(z) on the unit circle in the complex plane ( $z=e^{j2\pi \bar{f}}$ ), it is possible to derive the PSD of the analysed stationary random process Y exploiting spectral factorization:

$${\text{P}}_{\text{Y}}\left(\bar{f}\right)=H\left(\bar{f}\right)\Sigma_{{\text{U}}_{\text{Y}}}H^{*}\left(\bar{f}\right).$$ (29)

Importantly, spectral factorization is the core of the causal analysis of dynamic processes studied in the frequency domain and, while it is ubiquitously performed using AR modeling, could actually be obtained regardless of it (Baccalá and Sameshima, 2022). As specified in Section 1.2, the PSD matrix ${\text{P}}_{\text{Y}}\left(\bar{f}\right)$ contains information related to the spectral properties of the two processes, i.e., to their own dynamics, through the 2 diagonal elements $P_{Y_{i}}\left(\bar{f}\right)$ , i=1,2 , and to the causal interactions between $Y_{1}$ and $Y_{2}$ , through the off-diagonal elements $P_{Y_{i}Y_{j}}\left(\bar{f}\right)$ , i,j∈{1,2},i≠j .

The bivariate interactions between the processes $Y_{1},Y_{2}$ can be assessed by measures of spectral coupling and causality in the frequency domain, which can be directly derived from different combinations of the elements of the 2×2 PSD matrix ${\text{P}}_{\text{Y}}\left(\bar{f}\right)$ , of the 2×2 TF matrix $\text{H}\left(\bar{f}\right)$ and of the innovations of the full ARX and the restricted AR models. Under the assumption that the input white noises are uncorrelated at lag zero leading to diagonality of $\Sigma_{{\text{U}}_{\text{Y}}}$ (i.e., the model is strictly causal) (Ding et al., 2006; Faes et al., 2012a), from Equation 29 the $ij^{\text{th}}$ elements of ${\text{P}}_{\text{Y}}\left(\bar{f}\right)$ can be factorized into:

$$P_{Y_{i}Y_{j}}\left(\bar{f}\right)=\overset{2}{\underset{q=1}{\sum }}\sigma_{U_{Y_{q}}}^{2}H_{Y_{i}Y_{q}}\left(\bar{f}\right)H_{Y_{j}Y_{q}}^{*}\left(\bar{f}\right),$$ (30)

where $\sigma_{U_{Y_{q}}}^{2}$ , q=1,2 , are the diagonal elements of $\Sigma_{{\text{U}}_{\text{Y}}}$ . This factorization allows to decompose the frequency domain measures of coupling and causality into terms eliciting the directional information from one process to another. Indeed, exploiting Equation 30 the Coh between the two processes $Y_{1}$ and $Y_{2}$ Equation 5 is decomposed as:

$$\Gamma_{Y_{1};Y_{2}}\left(\bar{f}\right)=\overset{2}{\underset{q=1}{\sum }}\gamma_{Y_{1}Y_{q}}\left(\bar{f}\right)\gamma_{Y_{2}Y_{q}}^{*}\left(\bar{f}\right),$$ (31)

where $\gamma_{Y_{i}Y_{j}}\left(\bar{f}\right)$ is the so-called directed coherence (DC) (Saito and Harashima, 1981; Baccalá et al., 1998) quantifying frequency-specific causal influences from $Y_{j}$ to $Y_{i}$ ( i,i=1,2 ; the measure quantifies internal influences within a process when i=j ). In particular, the squared modulus of the DC from the driver process $Y_{1}$ to the target process $Y_{2}$ appearing in Equation 31 denoted also causal coherence (Porta et al., 2002a) and defined as:

$$|\gamma_{Y_{2}Y_{1}}\left(\bar{f}\right){|}^{2}=\frac{\sigma_{U_{Y_{1}}}^{2}|H_{Y_{2}Y_{1}}\left(\bar{f}\right){|}^{2}}{\sigma_{U_{Y_{1}}}^{2}|H_{Y_{2}Y_{1}}\left(\bar{f}\right){|}^{2}+\sigma_{U_{Y_{2}}}^{2}|H_{Y_{2}Y_{2}}\left(\bar{f}\right){|}^{2}},$$ (32)

can be interpreted as a measure of the influence of $Y_{1}$ onto $Y_{2}$ , being 0 in the absence of directional coupling from $Y_{1}$ to $Y_{2}$ at the frequency f , and 1 in the presence of full coupling. Importantly, if the bivariate process is strictly causal the squared DC $|\gamma_{Y_{2}Y_{1}}\left(\bar{f}\right){|}^{2}$ reflects the coupling strength from $Y_{1}$ to $Y_{2}$ intended as the normalized proportion of $P_{Y_{2}}\left(\bar{f}\right)$ which is causally due to $Y_{1}$ . This interpretation arises from the observation that, for strictly causal processes, Equation 30 written for the target process becomes $P_{Y_{2}}\left(\bar{f}\right)=P_{Y_{2}|Y_{1}}\left(\bar{f}\right)+P_{Y_{2}|Y_{2}}\left(\bar{f}\right)$ , where $P_{Y_{2}|Y_{1}}\left(\bar{f}\right)=\sigma_{U_{Y_{1}}}^{2}|H_{Y_{2}Y_{1}}\left(\bar{f}\right){|}^{2}$ is the numerator of the DC (Equation 32), the denominator being the whole spectrum $P_{Y_{2}}\left(\bar{f}\right)$ . Therefore $P_{Y_{2}|Y_{1}}\left(\bar{f}\right)=|\gamma_{Y_{2}Y_{1}}\left(\bar{f}\right){|}^{2}P_{Y_{2}}\left(\bar{f}\right)$ is the part of $P_{Y_{2}}\left(\bar{f}\right)$ due to $Y_{1}$ , which is usually referred to as the *causal* part of $P_{Y_{2}}\left(\bar{f}\right)$ ; $P_{Y_{2}|Y_{2}}\left(\bar{f}\right)=|\gamma_{Y_{2}Y_{2}}\left(\bar{f}\right){|}^{2}P_{Y_{2}}\left(\bar{f}\right)$ measures the part of $P_{Y_{2}}\left(\bar{f}\right)$ due to none of the other processes, but to the process $Y_{2}$ itself, which has been referred to as the *isolated* part of the target spectrum (Sparacino et al., 2024a). It is worth stressing that the DC (Equation 32) is a non-negative measure of causal coupling, but its meaningful physical interpretation as the amount of signal power transferred from the driver to the target process holds only under strict causality, because the denominator of (Equation 32) is not the PSD of the target process if instantaneous effects are present. To overcome this interpretational gap, extended versions of the causal coherence have been proposed in the literature (Faes and Nollo, 2010; 2013) which make use of AR models with instantaneous effects incorporated into the regression parameters.

Another line of research introduced independently frequency-domain measures of symmetric and directed interaction between two stationary jointly Gaussian processes based on the spectral expansion of the time-domain measures reviewed in Section 2.3) (Geweke, 1982; Ding et al., 2006; Chicharro, 2011). Given the spectral density matrix of the bivariate process, the frequency-specific measure of TD between $Y_{1}$ and $Y_{2}$ , $f_{Y_{1};Y_{2}}\left(\bar{f}\right)$ , was introduced as in Equation 6; the measure is null at the frequencies for which the two processes are uncoupled, and does not have an upper bound; it is indeed related to the magnitude squared coherence through Equation 7. Moreover, the measure satisfies the so-called *spectral integration property*, which relates the spectral measure (Equation 6) with the time-domain coupling measure (Equation 25) as follows (Gelfand and Yaglom, 1959):

$$F_{Y_{1};Y_{2}}=2\int_{0}^{\frac{1}{2}}f_{Y_{1};Y_{2}}\left(\bar{f}\right)\;\text{d}\bar{f}.$$ (33)

Moreover, exploiting the bivariate AR model and spectral factorization, Geweke formulated the so-called *linear feedback* measure defined as (Geweke, 1982)

$$f_{Y_{1}\to Y_{2}}\left(\bar{f}\right)=\log\frac{P_{Y_{2}}\left(\bar{f}\right)}{\sigma_{U_{Y_{2}}}^{2}|H_{Y_{2}Y_{2}}\left(\bar{f}\right){|}^{2}},$$ (34)

which is non-negative and linked to the corresponding time domain GC measure (Equation 27) by the spectral integration property according to a relation similar to that of Equation 33:

$$F_{Y_{1}\to Y_{2}}=2\int_{0}^{\frac{1}{2}}f_{Y_{1}\to Y_{2}}\left(\bar{f}\right)\;\text{d}\bar{f}.$$ (35)

The Geweke measure of spectral causality is an upper unbounded measure of GC which, if the bivariate process is strictly causal, can be related to the normalized measure of causal coherence. In fact, combining Equations 32, 34 one can easily show that the DC and the spectral GC are linked by the relation $f_{Y_{1}\to Y_{2}}\left(\bar{f}\right)=-\log\left(1-|\gamma_{Y_{2}Y_{1}}\left(\bar{f}\right){|}^{2}\right)$ (Geweke, 1982; Chicharro, 2011; Faes et al., 2012a; Pernice et al., 2022b; Sparacino et al., 2024a). We note that, while the TD measure (Equation 6) is always non-negative, the two causal measures $f_{Y_{i}\to Y_{j}}\left(\bar{f}\right)$ , i,j=1,2 , can take negative values at some frequencies if the process Y is not strictly causal (i.e., if the innovation covariance $\Sigma_{{\text{U}}_{\text{Y}}}$ is not diagonal) (Pernice et al., 2022b).

Finally, the spectral measure of *instantaneous causality* (IC) was chosen *ad hoc* as (Geweke, 1982)

$$f_{Y_{1}\cdot Y_{2}}\left(\bar{f}\right)=\log\frac{\sigma_{U_{Y_{1}}}^{2}|H_{Y_{1}Y_{1}}\left(\bar{f}\right){|}^{2}\sigma_{U_{Y_{2}}}^{2}|H_{Y_{2}Y_{2}}\left(\bar{f}\right){|}^{2}}{\left|{\text{P}}_{\text{Y}}\left(\bar{f}\right)\right|}$$ (36)

to satisfy the *Geweke decomposition of the total dependence* in the frequency domain, i.e.,

$$f_{Y_{1};Y_{2}}\left(\bar{f}\right)=f_{Y_{1}\to Y_{2}}\left(\bar{f}\right)+f_{Y_{2}\to Y_{1}}\left(\bar{f}\right)+f_{Y_{1}\cdot Y_{2}}\left(\bar{f}\right),$$ (37)

in such a way to be linked to the corresponding time domain IC measure (28) by the spectral integration property similarly to the relations established in Equations 33, 35 i.e.,

$$F_{Y_{1}\cdot Y_{2}}=2\int_{0}^{\frac{1}{2}}f_{Y_{1}\cdot Y_{2}}\left(\bar{f}\right)\;\text{d}\bar{f}.$$ (38)

The spectral measure defined in Equation 36 to satisfy Equation 37 and to be related to the time-domain measure of instantaneous causality by Equation 38 does not fulfil all the requirements of Geweke, different from what occurs in the time domain. Indeed, it may be negative for some frequencies and has no clear physical meaning (Geweke, 1982). In the absence of instantaneous causality, $F_{Y_{1}\cdot Y_{2}}=0$ because the prediction error covariance $\Sigma_{{\text{U}}_{\text{Y}}}$ is diagonal; however, $f_{Y_{1}\cdot Y_{2}}\left(\bar{f}\right)$ is generally found to be non-zero for some frequencies. Since the integral of $f_{Y_{1}\cdot Y_{2}}\left(\bar{f}\right)$ has to be null when $F_{Y_{1}\cdot Y_{2}}=0$ , not being zero for all frequencies, this implies the violation of non-negativity (Pernice et al., 2022b).

The issue of instantaneous causality in the computation of frequency domain measures of GC is a relevant one, and several efforts have been made to interpret instantaneous GC and to provide corrected measures (Faes and Nollo, 2010; Faes et al., 2013a; Nuzzi et al., 2021; Baccalá and Sameshima, 2021a). For instance, methods that identify a preferred direction for the instantaneous effects and then incorporate them together with lagged effects effects into directional measures of extended causality, were proposed by Faes and Nollo (2010) and Faes et al. (2013a): the methods exploit the Cholesky decomposition of the AR parameters and set the direction of zero-lag effects based on *a-priori* assumptions subjectively relying on physical knowledge (Faes and Nollo, 2010) or objectively relying on non-gaussianity of the AR residuals (Faes et al., 2013a). These methods were successfully applied to electroencephalographic rhythms (Faes et al., 2013a), as well as to cardiovascular and cerebrovascular oscillations (Pernice et al., 2022a). Measures which include instantaneous causality in the modelling approach are generally preferred because they enforce that zero-lag effects are ascribed to one of the two causal directions and therefore become zero both in time and frequency domain (Pernice et al., 2022a), essentially solving the issue about interpretability. Nevertheless, the assumptions about prior physiological knowledge or non-gaussianity of the residuals are not always satisfied, and therefore several alternatives based on keeping instantaneous effects as undirected but including them in extended spectral causality measures have been proposed to face the problem. As an example, Baccalá and Sameshima (2021a) discussed the theoretical interpretation of instantaneous GC within a spectral framework, and decomposed frequency-domain measures of causality, namely, directed transfer function and partial directed coherence, into lagged and instantaneous connectivity terms without the need of including the zero lag in AR models. On the other hand, Nuzzi et al. (2021) introduced an alternative frequency-domain decomposition of GC by obtaining a novel index of undirected instantaneous causality and a novel measure of GC including instantaneous effects, with the purpose to mitigate the confounding effect of zero-lag coupling. The issue of how it is best to treat instantaneous effects in the analysis of physiological interactions, e.g., cardiovascular interactions, where zero-lag interdependencies are expected to contribute significantly to the baroreflex mechanism (see, e.g., Faes et al. (2013a)), and of cardiorespiratory interactions, where the information transfer from respiration to heart rate variability is expected to be within-beat (Faes et al., 2012b), still remains an active area of research.

### 3.5 Connection between information-theoretic and spectral formulations

When formulated for jointly stationary Gaussian processes, the Geweke spectral and time domain measures of coupling and causality reviewed in the previous subsections have a straightforward information-theoretic interpretation (Geweke, 1982; Pernice et al., 2022b; Sparacino et al., 2024a). Indeed, the spectral measures of the bivariate interactions between two processes, defined by the spectral TD (Equation 6), the spectral GC (Equation 34) and the spectral IC (Equation 36), are closely related to the information-theoretic measures defined in Equations 11, 13, 15 by means of the spectral integration property (Geweke, 1982; Chicharro, 2011):

$$I_{Y_{1};Y_{2}}=\frac{F_{Y_{1};Y_{2}}}{2}=\int_{0}^{\frac{1}{2}}f_{Y_{1};Y_{2}}\left(\bar{f}\right)\;\text{d}\bar{f},$$ (39a)

$$T_{Y_{i}\to Y_{j}}=\frac{F_{Y_{i}\to Y_{j}}}{2}=\int_{0}^{\frac{1}{2}}f_{Y_{i}\to Y_{j}}\left(\bar{f}\right)\;\text{d}\bar{f};i,j=1,2,i\neq j$$ (39b)

$$I_{Y_{1}\cdot Y_{2}}=\frac{F_{Y_{1}\cdot Y_{2}}}{2}=\int_{0}^{\frac{1}{2}}f_{Y_{1}\cdot Y_{2}}\left(\bar{f}\right)\;\text{d}\bar{f}.$$ (39c)

The spectral integration property is very important not only to connect the information-theoretic and spectral formulations of the interaction measures, but also to allow quantification of these measures with reference to specific oscillatory components contained within spectral bands of interest. Examples of band-specific integration of the spectral interaction measures to obtain values related to peculiar oscillations of a group of random processes are reported in the next sections for real-world systems.

## 4 Implementation through model-free approaches

The practical implementation of the information-theoretic measures of symmetric and directional coupling through model-free approaches assumes that the measures are estimated directly from data without assuming a parametric model for the underlying probability distribution. These approaches are especially useful in high-dimensional, non-Gaussian, or complex distributions where classical parametric methods fail. This section presents three widely used model-free approaches for estimating entropy-based measures of coupling and causality, i.e., the k -nearest neighbour (Section 3.1), the binning (Section 3.2) and the permutation (Section 3.3) approaches. Other techniques, including kernel and slope estimators, are also discussed in the literature; however, they are not considered here for brevity reasons, and the reader is referred to Barà et al. (2024) for further details.

### 4.1 Nearest-neighbour estimator

The k -*nearest neighbour* (KNN) method is one of the most widely used entropy estimators (Ince et al., 2013; Wibral et al., 2013; Porta et al., 2016; Trujillo, 2019; Xiong et al., 2017; Azami et al., 2023), due to its ability to dynamically adjust resolution by adapting the distance scale to the underlying probability distribution (Victor, 2002), and its potential for bias correction through distance projection (Kraskov et al., 2004). This method builds upon the findings of Kozachenko and Leonenko (1987), who demonstrated that the average Shannon information of a d -dimensional random variable W can be approximated using the nearest neighbour distances among N observations $\left\{w_{1},w_{2},\ldots ,w_{N}\right\}$ . Specifically, the expectation of the log-probability of a sample point $w_{n}$ is estimated as:

$$-E\left[\log p\left(w_{n}\right)\right]=\psi \left(N\right)-\psi \left(k\right)+d\;\left\langle \log\varepsilon_{n}\right\rangle ,$$ (40)

where ψ(⋅) denotes the digamma function, and $\varepsilon_{n}$ is twice the distance (measured under the maximum norm) between $w_{n}$ and its $k^{\text{th}}$ nearest neighbour. The notation ⟨⋅⟩ indicates an average over the entire set of N samples. Then, for instance, based on Equation 40 the KNN estimation of the entropy of the present state of $Y_{1}$ can be expressed as:

$$H\left(Y_{1,n}\right)=\psi \left(N\right)-\psi \left(k\right)+\left\langle \log\varepsilon_{n}\right\rangle .$$ (41)

Besides the entropy of a scalar variable formulated as in Equation 41, the nearest neighbour estimator can be used to compute all of the entropy terms that compose a given coupling measure. However, as shown in Equation 13 for thr transfer entropy, the coupling and causality measures are expressed as combinations of entropy terms computed in spaces of different dimensions. Using the same nearest-neighbour search across these spaces leads to inconsistent distance scales, introducing estimation biases not canceled by entropy differences. To mitigate computational bias, the process begins by identifying the nearest neighbours within the full high-dimensional space, and then examining how these neighbours distribute across various lower-dimensional projections (Kraskov et al., 2004). Following this approach, given the bivariate system $\text{Y}=\left\{Y_{1},Y_{2}\right\}$ , the joint entropy over the combined space $\left[Y_{1,n},Y_{2,n},Y_{1,n}^{q},Y_{2,n}^{q}\right]$ , where q is the number of lags used to truncate the past of the processes, is first estimated as:

$$H\left(Y_{1,n},Y_{2,n},Y_{1,n}^{q},Y_{2,n}^{q}\right)=\psi \left(N\right)-\psi \left(k\right)+2\left(q+1\right)\left\langle \log\varepsilon_{n}\right\rangle ,$$ (42)

where $\varepsilon_{n}$ is twice the distance from $\left[y_{1,n},y_{2,n},y_{1,n}^{q},y_{2,n}^{q}\right]$ to its $k^{\text{th}}$ nearest neighbour. Then, the entropies in lower-dimensional subspaces are computed using the same radius $\varepsilon_{n}$ via range search. For the process $Y_{1}$ we obtain:

$$H\left(Y_{1,n},Y_{1,n}^{q}\right)=\psi \left(N\right)-\left\langle \psi \left(N_{Y_{1,n}Y_{1,n}^{q}}+1\right)\right\rangle +\left(q+1\right)\left\langle \log\varepsilon_{n}\right\rangle ,$$ (43a)

$$H\left(Y_{1,n}^{q}\right)=\psi \left(N\right)-\left\langle \psi \left(N_{Y_{1,n}^{q}}+1\right)\right\rangle +q\left\langle \log\varepsilon_{n}\right\rangle ,$$ (43b)

where $N_{Y_{1,n}Y_{1,n}^{q}}$ and $N_{Y_{1,n}^{q}}$ count neighbours within a distance $\varepsilon_{n}/2$ from $\left[y_{1,n},y_{1,n}^{q}\right]$ and $y_{1,n}^{q}$ , respectively. Analogously, for the process $Y_{2}$ we obtain:

$$H\left(Y_{2,n},Y_{2,n}^{q}\right)=\psi \left(N\right)-\left\langle \psi \left(N_{Y_{2,n}Y_{2,n}^{q}}+1\right)\right\rangle +\left(q+1\right)\left\langle \log\varepsilon_{n}\right\rangle ,$$ (44a)

$$H\left(Y_{2,n}^{q}\right)=\psi \left(N\right)-\left\langle \psi \left(N_{Y_{2,n}^{q}}+1\right)\right\rangle +q\left\langle \log\varepsilon_{n}\right\rangle ,$$ (44b)

with $N_{Y_{2,n}Y_{2,n}^{q}}$ and $N_{Y_{2,n}^{q}}$ defined in the same way. The joint entropy of the past states of both processes is estimated as:

$$H\left(Y_{1,n}^{q},Y_{2,n}^{q}\right)=\psi \left(N\right)-\left\langle \psi \left(N_{Y_{1,n}^{q}Y_{2,n}^{q}}+1\right)\right\rangle +2q\langle \log\varepsilon_{n}\rangle ,$$ (45)

where $N_{Y_{1,n}^{q}Y_{2,n}^{q}}$ counts neighbours within $\varepsilon_{n}/2$ from $\left[y_{1,n}^{q},y_{2,n}^{q}\right]$ . Finally, using the entropy expressions derived in Equations 42–45 and substituting them into Equation 13, the following estimator of TE is derived (Faes et al., 2015a; Pinto et al., 2025a):

$$T_{Y_{1}\to Y_{2}}=\left\langle \psi \left(N_{Y_{2,n}Y_{1,n}^{q}Y_{2,n}^{q}}+1\right)-\psi \left(N_{Y_{1,n}^{q}Y_{2,n}^{q}}+1\right)-\psi \left(N_{Y_{2,n}Y_{2,n}^{q}}+1\right)+\psi \left(N_{Y_{2,n}^{q}}+1\right)\right\rangle .$$ (46)

From Equation 46, the IC measure and the MIR can be computed as:

$$I_{Y_{1}\cdot Y_{2}}=\psi \left(k\right)+\left\langle \psi \left(N_{Y_{1,n}^{q}Y_{2,n}^{q}}+1\right)-\psi \left(N_{Y_{1,n}Y_{1,n}^{q}Y_{2,n}^{q}}+1\right)-\psi \left(N_{Y_{2,n}Y_{1,n}^{q}Y_{2,n}^{q}}+1\right)\right\rangle ,$$ (47a)

$$I_{Y_{1};Y_{2}}=\psi \left(k\right)+\langle \psi \left(N_{Y_{1,n}^{q}}+1\right)+\psi \left(N_{Y_{2,n}^{q}}+1\right)-\psi \left(N_{Y_{1,n}Y_{1,n}^{q}}+1\right)-\psi \left(N_{Y_{2,n}Y_{2,n}^{q}}+1\right)-\psi \left(N_{Y_{1,n}^{q}Y_{2,n}^{q}}+1\right)\rangle .$$ (47b)

The accuracy of entropy estimators can vary depending on both the data size and the chosen number of nearest neighbours ( k ). Specifically, smaller values of k yield more local estimates that capture subtle data structures but are more sensitive to noise. Conversely, larger values produce smoother, more stable estimates that may overlook fine-scale dynamics (Lombardi and Pant, 2016). Thus, selecting k requires balancing the trade-off between bias and variance, as thoroughly described in many previous works on the topic [see, e.g., Kugiumtzis (2013); Barà et al. (2023); Pinto et al. (2024); Barà et al. (2024); Pinto et al. (2025b)].

#### 4.1.1 Embedding procedures

Finding embedding vectors that adequately approximate the infinite-dimensional past states of the processes is a critical step in estimating information-theoretical measures using model-free approaches. When working with time series of finite length, e.g., the 300 samples typically used for the analysis of short-term physiological time series (Shaffer and Ginsberg, 2017), the employment of high-dimensional vectors to provide a more complete description of past processes leads to the curse of dimensionality and unreliable estimates of entropy quantities (Faes and Porta, 2014). A selection technique widely used in this frame is the *uniform embedding* approach, which simply uses a fixed number of equally spaced variables. Nevertheless, this method may overlook the most informative lags, potentially limiting the effectiveness of information-theoretic analyses in capturing relevant temporal dynamics (Kugiumtzis, 2013). An alternative approach was introduced to limit the size of the descriptive patterns and maximize their informational content about process dynamics, i.e., the *non-uniform embedding* approach introduced in (Faes et al., 2011; 2015a). In brief, a set of candidates including all possible states of the processes up to a maximum lag q is first considered. In the case of the TE estimation, with, e.g., $Y_{2}$ as the target process, a sequential approach is applied to fill progressively the embedding vector with the components taken from $\left[Y_{1,n}^{q},Y_{2,n}^{q}\right]$ which maximize the information shared with the present state of the target process $Y_{2,n}$ . Starting with an empty embedding vector, the additional information brought by each candidate above and beyond that provided by the previously selected variables is evaluated at each step, and the candidate bringing the maximum of such information is selected; the metric used to perform this maximization is the conditional MI. then, the selected candidate is retained and and added to the embedding vector if the contribution that it brings to the target is statistically significant; significance is typically assessed over randomized datasets obtained shuffling randomly the samples of the target series, and setting a threshold on the conditional MI based on percentiles. This step is repeated until no remaining candidate contributes a statistically significant amount of information (Faes et al., 2011; 2015a).

### 4.2 Binning estimator

The binning estimation approach is based on performing uniform or non-uniform quantization of a continuous random variable and then estimating the entropy of the variable approximating probabilities with the frequency of visitation of the quantized states, or bins. Specifically, uniform quantization simplifies implementation by dividing the range of values into equal intervals (Faes and Porta, 2014), whereas non-uniform quantization better preserves the dynamic structure of data by adapting to its distribution (Darbellay and Vajda, 1999).

Let W be a generic continuous random variable defined over the interval $D_{W}=\left[w_{\min},w_{\max}\right]$ . Quantization transforms W into a discrete random variable $B_{W}$ that assumes values from a finite alphabet $A_{B_{W}}=\left\{1,2,\ldots ,b\right\}$ , where b denotes the total number of quantization bins. In the case of uniform quantization, an observation $v\in D_{W}$ is assigned to bin $b_{w}=i$ if it satisfies the condition $w_{\min}+\left(i-1\right)r\leq v<w_{\min}+ir$ , where $r=\left(w_{\max}-w_{\min}\right)/b$ represents the uniform bin width (Azami et al., 2023; Barà et al., 2023). Following the quantization process, the probability associated with each symbol in the discrete alphabet $A_{B_{W}}$ is naturally approximated by its empirical frequency across a large number of observations, that is $\hat{p}\left(i\right)=\text{Pr}\;\left\{b_{w}=i\right\}=N_{i}/N$ , where $N_{i}$ is the number of observations of the variable that fall into the bin. The entropy of the original variable W can then be estimated by computing the entropy of the quantized variable $B_{W}$ by

$$H\left(W\right)=-\underset{b_{w}\in A_{B_{W}}}{\sum }\hat{p}\left(b_{w}\right)\log\hat{p}\left(b_{w}\right).$$ (48)

The concepts outlined above extend naturally to multivariate cases, where quantization is performed independently on each scalar element of the analysed vector variable. Specifically, if we consider a d -dimensional continuous random vector $W=\left[W_{1},\ldots ,W_{d}\right\}$ , each component can be discretized using b uniform bins; the resulting discrete vector variable $B_{W}=\left[B_{W_{1}},\ldots ,B_{W_{d}}\right]$ assumes values from a finite alphabet containing $b^{d}$ unique combinations of bin indices.

In the context of dynamic processes, the two stochastic processes $Y_{1}$ and $Y_{2}$ are represented by temporally-correlated vector-valued random variables. These vectors are formed by concatenating the current values with previous samples from both processes, extending up to q time steps into the past (Barà et al., 2023). The computation of TE and IC, as defined in Equations 13, 15, involves four entropy terms; therefore, Equation 48 can be used to estimate these measures. For TE, the required entropies correspond to the following vector configurations: (i) the past of the target process alone ( $b^{q}$ ); (ii) the past together with its current state ( $b^{q+1}$ ); (iii) the joint past of both processes ( $b^{2q}$ ); and (iv) the joint past combined with the present state of the target process ( $b^{2q+1}$ ). For IC, the entropy terms involve: (i) the joint past of both processes ( $b^{2q}$ ); (ii) the joint past with the present of one process ( $b^{2q+1}$ ); and (iii) the joint past with the present of both processes ( $b^{2q+2}$ ). Finally, the estimation of the MIR relies on the TE and IT estimators, according to the decomposition given in Equation 14.

A key issue in implementing discretization methods is the selection of the free parameters of the estimator, i.e., the memory length q used to capture the process history and, for the binning estimator, the number of quantization levels b used for coarse graining. These choices relate to estimating entropy in high-dimensional variables from limited data, tied to the curse of dimensionality (Runge et al., 2012). Empirically, parameter optimization aims to keep the alphabet size comparable to the series length N (Faes and Porta, 2014). Specifically, as regards the impact of the memory length, too small q implies that important dynamics or dependencies in the past are ignored, leading to underestimation of TE or MIR. On the other hand, too large q determines an increase of embedding dimension, such that the number of cells in the q-dimensional space grows exponentially ( $∼b^{q}$ ); in the case of small sample sizes this implies that the observations spread in the high-dimensional space, and most of the cells remain empty or sparsely populated, finally resulting in high variance of the estimates (the estimator becomes noisy and unreliable). The binning approach also requires a suitable choice of the number of quantization levels. Too small b leads to coarse quantization such that TE and MIR may be underestimated; conversely, too large b determines fine quantization, such that many bins have few or zero samples again resulting in high variance and possible overestimation due to noise [see, e.g., Barà et al. (2023), Barà et al. (2024)].

### 4.3 Permutation estimator

Permutation-based methods carry out symbolization by operating directly on discrete vector variables derived emphasizing the relative ordering of neighbouring sample amplitudes within each realization, rather than their specific absolute amplitude values (Bandt and Pompe, 2002). Given a general d− dimensional continuous random vector $W=\left[W_{1},\ldots ,W_{d}\right]$ , a discrete variable $R_{\text{W}}$ is derived by applying a rank-ordering transformation. For any realization $w=\left[w_{1},\ldots ,w_{d}\right]$ of W , the associated realization of $R_{\text{W}}$ is denoted by $r_{\text{W}}=\left[r_{w_{1}},\ldots ,r_{w_{d}}\right]\in A_{R_{\text{W}}}$ , where each $r_{w_{i}}\in \left\{1,\ldots ,d\right\}$ represents the rank position of $v_{i}$ when the elements of w are arranged in ascending order. For instance, $r_{w_{i}}=1$ if $w_{i}=\min\left(w\right)$ , and $r_{w_{i}}=d$ if $w_{i}=\max\left(w\right)$ . In the case of equal values, the element appearing later in the original sequence is assigned the smaller rank. After the discretization step, the probability associated with each symbol in the alphabet $A_{R_{\text{W}}}$ is estimated in a straightforward manner by computing its relative frequency across a large number of observations. Consequently, the entropy of the original continuous variable W is approximated by computing the entropy of its discretized counterpart $R_{\text{W}}$ , using a formulation similar to Equation 48 (Barà et al., 2023).

It is worth noting that the permutation strategy is favoured when compared with the binning approach for the estimation of entropy from a limited number of observations of the variable under analysis since for the first the continuous d -dimensional variable W takes values inside an alphabet with cardinality $\left|A_{R_{\text{W}}}\right|=d!$ , which is usually smaller than the cardinality of the alphabet obtained quantizing the variable with b bins, $\left|A_{B_{\text{W}}}\right|=b^{d}$ (Azami et al., 2023; Barà et al., 2023).

Analogously to the binning approach, estimation of the TE, presented in (Equation 13), requires that the relevant variables include either the past of the target process alone or combined with its present state, resulting in alphabet sizes of q! and (q+1)! , respectively. Additionally, the joint past of both processes is considered, with or without the past of the target, leading to alphabet sizes of ${\left(q!\right)}^{2}$ or q!⋅(q+1)! . IC, formulated in Equation 15, is estimated using combinations of the past and present values of $Y_{1}$ and $Y_{2}$ . The corresponding alphabet sizes are ${\left(q!\right)}^{2}$ for past-only combinations, q!⋅(q+1)! when one present state is included, and ${\left(\left(q+1\right)!\right)}^{2}$ when both are included. Finally, the MIR is estimated based on its decomposition into TE and IC, as given in Equation 14.

For the permutation approach, the memory length q controls the temporal resolution and sensitivity to dependencies. With increasing q the number of possible ordinal patterns grows factorially ( ∼q! ); with limited data, many patterns may never occur thus leading to high variance and noisy TE/MIR estimates. Again, a bias-variance trade-off is required to get reliable estimates [see, e.g., Barà et al. (2023), Barà et al. (2024)].

## 5 Practical computation of bivariate interaction measures

The practical computation of the information-theoretic and spectral measures of coupling and causality from two time series of L samples measured from a physical system, ${\text{y}}_{i}=\left\{y_{i}\left(1\right),\ldots ,y_{i}\left(L\right)\right\}$ , where i=1,2 and L is the length of the time series, is based on considering the series as a finite length realization of the vector process $\text{Y}=\left\{Y_{1},Y_{2}\right\}$ that describes the evolution of the system over time.

The software and the codes relevant to this work are collected in the BIM Matlab toolbox and available for free download from https://github.com/LauraSparacino.

### 5.1 Non-parametric estimation of the PSD

Spectral measures of coupling can be computed directly from the PSD terms of the set $\text{y}=\left\{{\text{y}}_{1},{\text{y}}_{2}\right\}$ as evidenced in Section 1.2; time domain and information-theoretic measures of dynamic coupling can then be obtained exploiting the spectral integration property applied to the spectral estimates under the hypothesis of linearity (see Section 2.4 and Section 2.5).

A widely used *non-parametric* estimator of the PSD is the weighted covariance (WC) method (function bim_WCspectra.m). This estimator leverages the FT of the sample CCF of the observed data ${\hat{R}}_{{\text{y}}_{1}{\text{y}}_{2}}\left(k\right)$ (Blackman and Tukey, 1958) and is expressed as:

$${\hat{P}}_{{\text{y}}_{1}{\text{y}}_{2}}\left(\bar{f}\right)=\overset{\tau }{\underset{k=-\tau }{\sum }}w\left(k\right)\;{\hat{R}}_{{\text{y}}_{1}{\text{y}}_{2}}\left(k\right)\;e^{-\text{j}2\pi \bar{f}k},$$ (49)

where τ≤L−1 is the maximum lag considered for estimating the CCF. The window function w(k) has width 2τ , satisfies w(k)=0 for |k|>τ , is normalized such that 0≤w(k)≤w(0)=1 , and is symmetric, i.e., w(−k)=w(k) (Kay, 1988). To ensure non-negative spectral estimates as a result of Equation 49, it is common to use biased estimators for the CCF producing semi-definite sequences. A biased estimator is given by:

$${\hat{R}}_{{\text{y}}_{1}{\text{y}}_{2}}\left(k\right)=\frac{1}{L}\overset{L-1-k}{\underset{n=0}{\sum }}{\text{y}}_{1,n}^{*}\;{\text{y}}_{2,n-k},$$ (50)

where the asterisk denotes the complex conjugate. The definition in Equation 50 applies for k=0,…,L−1 , while for negative lags k=−(L−1),…,−1 , the cross-correlation is defined by symmetry as ${\hat{R}}_{{\text{y}}_{1}{\text{y}}_{2}}\left(k\right)={\hat{R}}_{{\text{y}}_{1}{\text{y}}_{2}}^{*}\left(-k\right)$ . The same reasoning applies to the computation of the autocorrelation function ${\hat{R}}_{{\text{y}}_{i}}\left(k\right)$ to get the autospectrum ${\hat{P}}_{{\text{y}}_{i}}\left(k\right)$ , with i=1,2 . Window selection is usually performed by providing mathematical formulations for the window function which allow to control the spectral leakage introduced by the profile of the window itself (Pinna et al., 1996). Following this rationale, the Parzen window can be suitably selected, since it shows a significantly lower side-lobe level compared to Hanning and Hamming windows; furthermore, it is non-negative for all frequencies, and produces non-negative spectral estimates (Priestley, 1981). For the Parzen window, the relationship between the bandwidth ( $B_{w}$ ) of the spectral window and the lag τ at which correlation estimates are truncated is $B_{w}=1.273f_{s}/\tau$ (Pinna et al., 1996). To resolve the corresponding peaks in the spectrum, the window bandwidth can be set equal to 25 Hz, which brings to τ≈0.05 using $f_{s}=1$ Hz.

### 5.2 Linear autoregressive models

The linear ARX equation in Equations 16a,b is seen as a model of how the observed data have been generated, and an identification procedure (function bim_idARX.m) is applied after model order selection (function bim_mos_idARX.m) to provide estimates of the coefficients and innovation variances to be used for computing the coupling and causality measures in the information-theoretic and spectral domains. Then, computation of the information-theoretic measures of MIR, TE and IT amounts to identify restricted linear models through methods which extract the parameters of the restricted model from those of the full model (Section 2.2), i.e., based on (i) the resolution of the YW equations (function bim_MIRdec_lin_YW.m) or, equivalently, on (i) SS models (function bim_MIRdec_lin_SS.m). Side functions of (i) are bim_LinReg.m, which performs a linear regression of the present state of input target processes from the past states of input driver processes, and bim_Yule.m, which provides solution of the YW equations for a ARX process using discrete time Lyapunov equation; side functions of (ii) are bim_SSmodel.m, which computes the parameters of a SS model from those of a ARX model, and bim_submodel.m, which derives a submodel of a SS model. On the other hand, information-theoretic measures of coupling and causality can be obtained directly exploiting the spectral integration property (Section 2.4 and Section 2.5). If this is the case, *spectral* measures of TD, GC and IC are computed by first estimating the parametric PSD matrix of the set $\left\{{\text{y}}_{1},{\text{y}}_{2}\right\}$ (function bim_VARspectra.m). Then, the function bim_fGC_lin.m computes the spectral measures, accepting as inputs the PSD of the process, the transfer function of the full model and the innovation variances. The information-theoretic counterparts are finally obtained as the integrals of the corresponding spectral functions up to a factor 2.

### 5.3 Model-free approaches

Model-free estimation of the MIR, TE and IT measures can be performed by exploiting different approaches for the computation of entropy measures (Xiong et al., 2017; Azami et al., 2023). Regarding the approaches reviewed in this work, the KNN, binning and permutation estimators are implemented through the functions bim_MIRdec_KNN.m, bim_MIRdec_bin.m and bim_MIRdec_perm.m, respectively, taking as inputs the set $\left\{{\text{y}}_{1}^{⊺},{\text{y}}_{2}^{⊺}\right\}$ in the form of a L×2 matrix, the number of past samples in the embedding vectors, and the vector of embedding delays τ . Further, the number of neighbours and quantization bins must be specified for the KNN and binning estimators, respectively. Side functions are bim_SetLag.m, which returns the list of candidates taking as input the vectors of embedding dimensions and delays (uniform embedding), bim_quantization.m, which quantizes the input series with a given number of quantization levels (for binning estimator), and bim_H.m, which computes entropy of a discrete multidimensional variable. The example bim_thsim_modelfree.m replicates physiological interactions and shows the behaviour of the three estimators at varying hyperparameters such as memory length, quantization levels, or the number of nearest neighbors.

### 5.4 Assessment of statistical significance

This section presents the use of *surrogate data analysis* to statistically validate the proposed measures of coupling and causality in the information-theoretic and spectral domains. Validation is performed at the level of individual realizations of the observed random processes $\left\{Y_{1},Y_{2}\right\}$ , obtained in the form of the set of time series ${\text{y}}_{i}$ , i=1,2 .

The method of surrogate data (Theiler et al., 1992; Zhang, 2023) is employed to set a significance level for the coupling and causality measures. Specifically, to assess the significance of conditional mutual information measures (i.e., $T_{Y_{i}\to Y_{j}}$ and $I_{Y_{i}\cdot Y_{j}}$ , i,j=1,2,i≠j and/or their Gaussian logarithmic counterparts - see Section 2.5), as well as of the MIR/TD, it is sufficient to destroy the coupling of the two series, while it is preferable to maintain the statistical properties of the individual series. Therefore, *random time shift bivariate surrogates* are generated according to the null hypothesis of independent random processes, shifting the samples of the time series $Y_{1}$ over time (while wrapping the extra values around the beginning of the series) and leaving the other series unchanged; the shift is chosen randomly, imposing a minimum shift of $\tau_{\min}$ lags (function bim_surrtimeshift.m). For each pair of original time series, $n_{s}$ pairs of surrogate time series are generated to obtain the set ${\text{y}}_{i}^{s}$ ( $i=1,2;s=1,\ldots ,n_{s}$ ). The considered measure is then computed both on the original series and on each surrogate pair $\left\{{\text{y}}_{1}^{s},{\text{y}}_{2}^{s}\right\}$ , yielding the surrogate distribution from which the significance threshold is derived taking the $100{\left(1-\alpha \right)}^{\text{th}}$ percentile. Then, each conditional mutual information measure is deemed as statistically significant if its value computed on the original series is higher than the significance threshold. The same procedure applies to both information-theoretic and spectral measures, the latter obtained by integrating the spectral profiles within specific frequency bands. The reader is referred to (Pinto et al., 2024) for methodologies and codes relevant to the assessment of coupling and nonlinearity in bivariate time series.

## 6 Exemplary applications to real-world time series

### 6.1 Cardiovascular interactions

In this section, we analyse physiological time series collected to study the effect of postural stress on cardiovascular variability (Faes et al., 2013c; Bari et al., 2016). One representative subject was selected for the following analyses, chosen from a dataset comprising healthy controls enrolled at the Neurology Division of Sacro Cuore Hospital, Negrar, Italy. Electrocardiogram (ECG) was acquired synchronously with arterial pressure (AP) measured at the level of middle finger through a photopletysmographic device (Finapres Medical Systems, Ohmenda, Netherlands) at a sampling rate of 1 kHz. From the raw signals, stationary time series of heart period (*H*) and systolic AP (*S*) were measured as detailed in (Faes et al., 2013c; Bari et al., 2016) during the supine resting state condition, and regarded as realizations of the *H* and *S* discrete-time processes, in turn assumed as uniformly sampled with a sampling frequency equal to the inverse of the mean heart period. The series, each of length equal to 251 beats, were preprocessed reducing the slow trends with an AR high-pass filter (zero phase; cut-off frequency 0.0156 Hz), removing the mean value and normalizing to unit variance.

Information and spectral measures of coupling (MIR/TD) and causality (TE/GC, IT/IC) were computed from the parameters of an ARX model (least squares estimation, model order set according to the AIC - maximum scanned model order: 8; selected model order: 6), restricted through resolution of the YW equations and represented in the frequency domain to get a parametric estimation of the PSD matrix. The mathematical derivations are presented in Section 2, while the practical computation is detailed in Section 4. The spectral profiles were integrated within two frequency bands of physiological interest, i.e., the low frequency (LF, f∈[0.04−0.15] Hz) and the high frequency (HF, f∈[0.15−0.4] Hz) band, as well as over the whole frequency range ( $f\in \left[0-f_{s}/2\right]$ Hz) to get the corresponding time domain values (see Section 2.4 and Section 2.5). In heart rate variability analysis, the LF band reflects both sympathetic and parasympathetic activity, while the HF band indicates parasympathetic (vagal) influence, often linked to respiration (Task Force of the European Society of Cardiology and the North American Society of Pacing and Electrophysiology, 1996). Surrogate data analysis was applied as in Section 4.1 to assess the statistical significance of the computed measures, with a minimum shift of $\tau_{\min}=20$ lags, $N_{s}=100$ iterations and α=0.05 significance level.

**FIGURE 2:**
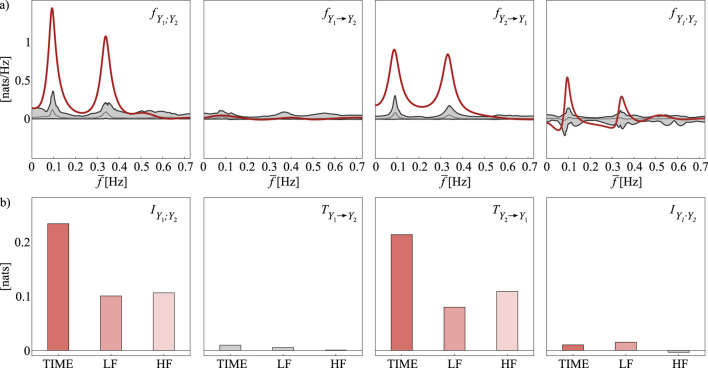
Cardiovascular signals show coherent oscillations in spectral bands with physiological meaning. **(a)** Red solid lines: spectral TD ( $f_{Y_{1};Y_{2}}$ ), GC ( $f_{Y_{1}\to Y_{2}}$ , $f_{Y_{2}\to Y_{1}}$ ) and IC ( $f_{Y_{1}\cdot Y_{2}}$ ) profiles, where $Y_{1}=H,Y_{2}=S$ . The surrogate distributions of the spectral profiles are depicted as shaded grey areas, median (grey solid lines) and percentiles (black solid lines, computed with 5% significance level). **(b)** MIR ( $I_{Y_{1};Y_{2}}$ ), TE ( $T_{Y_{1}\to Y_{2}}$ , $T_{Y_{2}\to Y_{1}}$ ) and IT ( $I_{Y_{1}\cdot Y_{2}}$ ) values integrated in the whole band (TIME, left bars), the low frequency (LF) band (middle bars) and the high frequency (HF) band (right bars) of the spectrum. Grey bars indicate non significant values according to surrogate data analysis.

Figure 2a displays the spectral profiles of TD ( $f_{Y_{1};Y_{2}}$ , with $Y_{1}=H,Y_{2}=S$ ), GC ( $f_{Y_{1}\to Y_{2}}$ and $f_{Y_{2}\to Y_{1}}$ ), and IC ( $f_{Y_{1}\cdot Y_{2}}$ ) - red line - along with their surrogate distributions - gray lines and shades. Figure 2b) depicts the corresponding information-theoretic domain measures, obtained by integrating the spectral measures in a) over the TIME ( $0-f_{s}/2$ ), LF ( 0.04−0.15 Hz) and HF ( 0.15−0.4 Hz) bands as in Equations 39a–39c. As regards the total coupling (panel a, left), two peaks are observed at ∼0.1 Hz and ∼0.35 Hz, corresponding to significant MIR values in the LF and HF bands, respectively (panel b, left). These findings are consistent with previous studies confirming the presence of LF oscillatory rhythms in heart rate and blood pressure variability, as well as the dominant role of HF spectral components of the respiratory signal during supine rest [see, e.g., Pagani et al. (1986); Montano et al. (1994); Cooley et al. (1998); Stefanovska (2002)]. The observed coupling primarily occurs in the direction $Y_{2}\to Y_{1}$ , that is, from *S* to *H*, rather than in the reverse direction (panels a,b, middle). This is supported by the low and statistically non-significant values of both the frequency domain measure $f_{Y_{1}\to Y_{2}}$ and the time domain measure $T_{Y_{1}\to Y_{2}}$ . In contrast, $f_{Y_{2}\to Y_{1}}$ exhibits two distinct peaks in the LF and HF bands, corresponding to sympathetic and vagal activity, respectively. The instantaneous interactions are predominantly concentrated in the LF band, as indicated by significance of the IT measure $I_{Y_{1};Y_{2}}$ (panel b, right). Although the spectral profile $f_{Y_{1}\cdot Y_{2}}$ (panel a, right) also shows a peak in the HF band, the integrated contribution over this band is not statistical significant. This behaviour is expected, as systolic arterial pressure and heart period are known to interact at zero lag due to shared physiological mechanisms such as the baroreflex and central autonomic regulation. This phenomenon has been documented in several studies (e.g., Porta et al., 2000; Nollo et al., 2005), supporting the interpretation that instantaneous causality between these cardiovascular parameters reflects genuine physiological coupling rather than non-physiological factors (e.g., unobserved confounders) (Faes et al., 2013a; Nuzzi et al., 2021).

### 6.2 Case study in climate science

In this section, to showcase the use of the tools presented in this paper also outside the field of Network Physiology, we consider an exemplary case study in climate science, i.e., we investigate the interactions among the most representative indices descriptive of El Niño and the Southern Oscillation (ENSO). ENSO is a periodic fluctuation in the sea surface temperature and air pressure of the atmosphere overlying the equatorial Pacific Ocean, which is considered as the most prominent interannual climate variability on Earth (McPhaden et al., 2006). Since the exact initiating causes of an ENSO warm or cool events are not fully understood, it is important to analyze the statistical relationship between its two main components, i.e., atmospheric pressure and sea surface temperature. Such components are measured respectively by NINO34 (the East Central Tropical Pacific sea surface temperature anomaly, also called El Niño) and SOI (Southern Oscillation Index, the standardized difference in surface air pressure between Tahiti and Darwin), and are dynamically related to several other indexes that represent large scale climate patterns (Chang et al., 2003; Silini et al., 2023; Pinto et al., 2025c). The analyzed climate indices are taken from a public database (Silini et al., 2023), of which we consider the series SOI and NINO34 measured with a monthly sampling rate during the period 1950–2016 (792 data points) for which all time series values are available. The series were first detrended and deseasonalized.

Model-based and model-free information-theoretic measures of MIR, TE and IT were computed exploiting the linear parametric, KNN, binning and permutation approaches. Specifically, linear parametric measures were computed from the parameters of an ARX model (least squares estimation, model order set according to the AIC - maximum scanned model order: 12 (Faes et al., 2012a); selected model order: 10); restricted models were obtained via resolution of the YW equations (Section 2). The KNN estimator was implemented through the uniform embedding procedure, by fixing a maximum number of past samples in the embedding vectors of q=3 samples, while a number of neighbours k=10 was set to estimate the information measures of coupling and causality (Section 3.1). As regards the binning approach, we set b=3 and q=2 , so as to deal with a number of quantization levels equal to $3^{2\times 2+2}$ and adhere with the empirical rule stated in Section 3.2. On the other hand, we used the pattern length typically adopted in permutation entropy analyses when the permutation approach was implemented, which is the minimum advised to guarantee variability in the discrete patterns, i.e., q=3 (Section 3.3). For all the non-linear estimators, the embedding delay was set equal to τ=3 . All the procedures are detailed in Section 4. Surrogate data analysis was applied as in Section 4.1 to assess the statistical significance of the computed measures, with a minimum shift of $\tau_{\min}=20$ lags, $N_{s}=100$ iterations and α=0.05 significance level.

**FIGURE 3:**
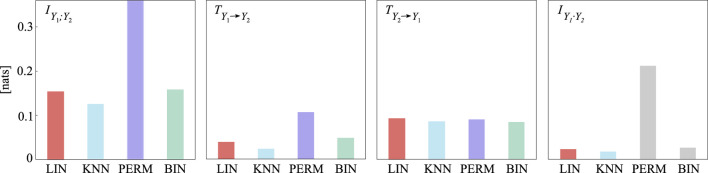
Different estimation approaches of coupling and causality measures suggest a bidirectional transfer of information in representative climate time series. The time domain MIR ( $I_{Y_{1};Y_{2}}$ ), TE ( $T_{Y_{1}\to Y_{2}}$ , $T_{Y_{2}\to Y_{1}}$ ) and IT ( $I_{Y_{1}\cdot Y_{2}}$ ) measures are estimated exploiting the linear parametric (LIN), *k*-nearest neighbours (KNN), permutation-based (PERM) and binning (BIN) approaches, with $Y_{1}=SOI,Y_{2}=NINO34$ . Grey bars indicate non significant values according to surrogate data analysis.

Figure 3 summarizes the coupling and causality measures of MIR ( $I_{Y_{1};Y_{2}}$ , with $Y_{1}=SOI,Y_{2}=NINO34$ ), TE ( $T_{Y_{1}\to Y_{2}}$ and $T_{Y_{2}\to Y_{1}}$ ) and IT ( $I_{Y_{1}\cdot Y_{2}}$ ) computed for a representative pair of climate time series estimated using the linear parametric (LIN) and three different model-free approaches, i.e., the KNN, permutation (PERM) and binning (BIN) approaches. Gray bars indicate non significant values according to surrogate data analysis. Significant dynamic coupling is detected between the two processes. The same behaviour is observed for the TE in both directions (middle), that is $T_{Y_{1}\to Y_{2}}$ and $T_{Y_{2}\to Y_{1}}$ . Finally, as regards the instantaneous transfer, only the LIN and KNN methods yield statistically significant results, although the values are very low and close to zero ( $I_{\text{LIN}}=0.022773$ nats; $I_{\text{KNN}}=0.017997$ nats), probably suggesting that the *NINO34* and *SOI* time series do not exhibit meaningful instantaneous information exchange. Rather, their interaction is likely dominated by delayed, feedback-driven dynamics typical of coupled ocean-atmosphere processes such as ENSO. This aligns with previous studies highlighting the temporal structure and lagged dependencies in ENSO-related indices, where causality is more evident over seasonal timescales than at zero lag (Faes et al., 2025b).

Overall, although the LIN, KNN, PERM and BIN estimators aim to quantify the same underlying information flow, their varying theoretical assumptions (continuous- or discrete-valued random variables), data manipulation steps (no transformation, discretization based on quantization or based on ordinal patterns), estimation approach (model-based vs. model-free), as well as their different sensitivity to data length, noise, and embedding parameters, explain why distinct numerical values may arise, even substantial as depicted in Figure 3. As a matter of fact, estimating information-theoretic measures such as transfer entropy or mutual information rate yields results that depend strongly on how probability distributions are inferred or system dynamics are modelled. Therefore, comparison between measures derived from different approaches should be avoided. Moreover, given an estimation approach, the parameter setting should be as much as possible uniform when comparing the same measure across different experimental conditions. In general, the selection of the appropriate estimator should be guided by the nature of the data, including its linearity, stationarity, noise characteristics, and available sample size.

## 7 Conclusion

This work provides a comprehensive review, theoretical description and practical implementation of the most popular time-domain, spectral and information-theoretic approaches for the investigation of both symmetrical and directional interactions in bivariate time series. Coupling and causality measures are described in their formulation, evaluated critically highlighting advantages and limitations, connected identifying their reciprocal relations, and showcased in exemplary applications in Network Physiology and Climate Science. Thanks to the freely available toolbox that practically implements the measures using model-based and model-free estimators, our groundwork provides researchers with a robust foundation for quantifying and interpreting several bivariate interdependencies across a wide range of applications.

The practical implementation of the coupling and causality measures through both model-based and model-free approaches allows a complete characterization of the complex interplay occurring in a variety of real-world scenarios. We show how parametric modelling offers interpretability and computational efficiency under the assumption of joint Gaussianity of the bivariate process analysed, and that fully model-free estimation techniques, including binning, permutation and nearest-neighbours estimators, achieve a non-linear description of the complex interdependencies among the data. Although inherently model-free, information-theoretic measures are herein contextualized through linear model-based interpretations, which enable frequency-specific insights into oscillatory dynamics.

The present work can thus serve as a driving force for future endeavours in the development and critical assessment of functional connectivity measure in bivariate systems, as well as help researchers to test such measures in a variety of applicative scenarios where the activity of dynamic systems is measured in terms of time series. Moreover, the systematic description and categorization of bivariate measures pursued in this work can pose solid basis to extend them to multivariate time series data, in contexts where more than two dynamic processes are simultaneously monitored. This approach is very popular in the field of Network Physiology, historically regarding the reconstruction of causal networks (Günther et al., 2022) where a range of extensions of the methods reviewed here has been proposed in the multivariate setting (Rosenberg et al., 1989; Baccalá and Sameshima, 2001; Faes et al., 2012b; Barnett and Seth, 2014; Montalto et al., 2014; Baccalá and Sameshima, 2021b; Baccalá and Sameshima, 2022), and more recently regarding the study of high-order interactions (Scagliarini et al., 2023) whereby collective interactions among three or more processes which cannot be reduced to pairwise dependencies are increasingly investigated extending tools for bivariate analysis (Faes et al., 2022; Scagliarini et al., 2023; Faes et al., 2025b; Sparacino et al., 2025b; Mijatovic et al., 2025; Faes et al., 2025a). These causal and high-order analyses of multivariate time series are closely connected (see, e.g., Stramaglia et al. (2024)), and their practical implementation implies to face similar issues to those tackled by the bivariate methods presented here, even though exacerbated by the need to work in higher-dimensional settings. In fact, as dimensionality increases, the joint probability space expands rapidly, raising theoretical constraints, computational demands and the risk of biased estimation, particularly with limited data. Up to now, while multivariate linear methods applied appear feasible across time, frequency and information-theoretic domains, truly multivariate non-parametric approaches to the study of causality networks and high-order interactions are scarce. The extension of the bivariate model-free methods reviewed here will have to face the theoretical and practical challenges posed by the curse of dimensionality, and is an open question for the ongoing research in the field.

## Data Availability

Publicly available datasets were analyzed in this study. This data can be found here: https://github.com/LauraSparacino.
